# Effects of Rosmarinic Acid and Doxorubicin Combination in Breast Cancer Cells

**DOI:** 10.3390/biology15131022

**Published:** 2026-06-26

**Authors:** Coşkun Orhaner, Aylin Orhaner, Mehmet Cudi Tuncer, İlhan Özdemir

**Affiliations:** 1 Department of Gynecology and Obstetrics, Soranus IVF Center, 16070 Bursa, Turkey; orhanercoskun@gmail.com; 2 Department of Gynecology and Obstetrics, Medicana Bursa Hospital, 16150 Bursa, Turkey; draylinorhaner@hotmail.com; 3 Department of Anatomy, Faculty of Medicine, Dicle University, 21280 Diyarbakır, Turkey; 4 Department of Histology and Embryology, Faculty of Medicine, Kahramanmaraş Sütçü İmam University, 46000 Kahramanmaras, Turkey; ilhanozdemir32@hotmail.com

**Keywords:** 4T1, apoptosis, breast cancer, doxorubicin, HaCaT, rosmarinic acid

## Abstract

Rosmarinic acid (RA), a naturally occurring polyphenolic compound, has demonstrated promising anticancer activity; however, its combinatorial potential with conventional chemotherapeutic agents remains incompletely characterized. This study investigated the cytotoxic, pro-apoptotic, oxidative stress-associated, and cytokine-associated effects of RA alone and in combination with doxorubicin (DOX) in 4T1 murine breast cancer cells and HaCaT human keratinocyte cells as a non-cancerous control model. Cellular viability, apoptosis, cell cycle progression, oxidative stress, mitochondrial function, cytokine responses, and apoptosis-associated molecular alterations were evaluated using complementary cellular and molecular approaches. In addition, three-dimensional (3D) tumor spheroid experiments were performed to assess treatment responses under physiologically relevant tumor-like conditions. Results demonstrated that RA synergistically enhanced DOX-induced cytotoxicity in 4T1 cells while exhibiting comparatively lower toxicity toward HaCaT cells. Combination treatment significantly increased apoptotic cell death, mitochondrial depolarization, intracellular reactive oxygen species (ROS) accumulation, apoptotic DNA fragmentation, and G_2_/M-phase accumulation. N-acetylcysteine (NAC)-mediated rescue experiments partially reversed ROS elevation and treatment-associated cytotoxicity in both monolayer and 3D spheroid models. Furthermore, the RA+DOX combination markedly disrupted spheroid integrity and reduced spheroid viability compared with monotherapies. Collectively, these findings indicate that RA enhances the anticancer activity of DOX and support further investigation of this combination strategy in breast cancer models.

## Simple Summary

Triple-negative breast cancer is an aggressive cancer subtype associated with poor prognosis, high metastatic potential, and limited therapeutic options. In the present study, the combined effects of rosmarinic acid and doxorubicin were investigated in 4T1 breast cancer cells using both two-dimensional and three-dimensional experimental models. The combination treatment reduced cell viability, increased apoptosis, altered cell cycle progression, and disrupted spheroid integrity more effectively than either treatment alone. Increased oxidative stress and apoptosis-associated molecular alterations were also observed following combined treatment exposure. Additional experiments using N-acetylcysteine indicated that oxidative stress contributes to the observed anticancer effects, although additional mechanisms are also likely involved. Overall, the findings suggest that rosmarinic acid may enhance the anticancer activity of doxorubicin through multifactorial biological responses. These results provide preliminary experimental evidence supporting further investigation of this combination strategy in future preclinical studies.

## 1. Introduction

Breast cancer remains the most common malignancy among women worldwide and continues to represent a major cause of cancer-related morbidity and mortality. According to 2022 global cancer statistics, the highest incidence of breast cancer was reported in Asia, with approximately 985,817 newly diagnosed cases, and the global disease burden is projected to exceed 6 million cases by 2050. Among breast cancer subtypes, triple-negative breast cancer (TNBC) is characterized by the absence of estrogen receptor (ER), progesterone receptor (PR), and human epidermal growth factor receptor 2 (HER2) expression and is considered one of the most aggressive and therapeutically challenging subtypes due to its poor prognosis and limited responsiveness to endocrine-targeted therapies [1]. The 4T1 murine breast cancer cell line is widely used as an experimental TNBC model because it closely mimics the aggressive and metastatic characteristics of human TNBC in vitro and in vivo [2,3,4].

DOX, a broad-spectrum anthracycline widely used in breast cancer treatment, exerts potent cytotoxic effects primarily through DNA intercalation and topoisomerase II inhibition. However, the clinical application of DOX is substantially limited by cumulative dose-dependent toxicities, including cardiotoxicity, myelosuppression, hepatotoxicity, nephrotoxicity, and gastrointestinal adverse effects. In particular, cardiotoxicity has been closely associated with ROS generation, mitochondrial dysfunction, and disruption of cellular signaling pathways [5]. In addition, prolonged DOX exposure contributes to the development of multidrug resistance (MDR), thereby reducing long-term therapeutic efficacy [6]. To overcome these limitations, increasing attention has been directed toward the use of natural bioactive compounds in combination with conventional chemotherapeutic agents [7].

RA is a naturally occurring polyphenolic ester predominantly found in medicinal and aromatic plants belonging to the Lamiaceae family, including rosemary (*Rosmarinus officinalis*), basil (*Ocimum basilicum*), sage (*Salvia officinalis*), and mint (*Mentha* spp.). RA has attracted considerable interest because of its antioxidant, anti-inflammatory, and anticancer properties. Previous studies have demonstrated that RA modulates several cancer-associated biological processes, including apoptosis, oxidative stress, inflammation, and cell cycle regulation [8,9]. RA has also been reported to exert antiproliferative and pro-apoptotic effects in various cancer models through regulation of oxidative stress-associated signaling pathways and apoptosis-related molecular mechanisms [10].

Previous investigations evaluating RA in combination with DOX have suggested that this combination may enhance anticancer activity. In ovarian adenocarcinoma models, RA has been shown to inhibit cellular proliferation, migration, and invasion while increasing apoptotic cell death and enhancing DOX sensitivity [11,12]. In addition, RA has been reported to suppress MDR1 transcription through downregulation of P-glycoprotein (P-gp) expression, thereby partially reversing multidrug resistance mechanisms in DOX-resistant cancer cells [13]. Experimental evidence further suggests that RA may attenuate DOX-associated cardiotoxicity through modulation of apoptosis-related pathways and cardiac remodeling processes [14]. Moreover, RA has been reported to alleviate DOX-induced oxidative stress, cytotoxicity, and genotoxicity in non-cancerous cellular models, suggesting a potential protective effect against chemotherapy-associated toxicity in healthy tissues [15]. In this context, the HaCaT human keratinocyte cell line is commonly used as a non-cancerous control model for comparative in vitro evaluations [6].

The intrinsic mitochondrial apoptotic pathway represents one of the principal mechanisms underlying chemotherapy-induced cancer cell death and involves disruption of mitochondrial membrane potential (ΔΨm), alterations in the Bax/Bcl-2 balance, cytochrome c release, and subsequent activation of caspase-9 and caspase-3 [16]. In addition to apoptosis-related signaling, oxidative stress and cell cycle dysregulation have also emerged as critical contributors to treatment-associated cytotoxicity. Functional characterization of these molecular responses is important for understanding the therapeutic potential of combination-based anticancer strategies. Furthermore, three-dimensional (3D) tumor spheroid systems have increasingly been recognized as physiologically relevant in vitro models that better recapitulate tumor architecture, diffusion gradients, and treatment responses compared with conventional monolayer cultures.

In the present study, the cytotoxic, apoptosis-associated, oxidative stress-associated, and cytokine-associated effects of RA, DOX, and the RA+DOX combination were comprehensively investigated using 4T1 murine breast cancer cells and HaCaT human keratinocyte cells [17]. Cell viability, apoptosis, cell cycle distribution, mitochondrial membrane potential, intracellular ROS accumulation, cytokine profiles, apoptosis- and cell cycle-related gene expression, immunocytochemical alterations, and NAC-mediated antioxidant rescue responses were evaluated using both monolayer and 3D tumor spheroid models. Although previous studies have reported selected biological effects of the RA+DOX combination, a comprehensive evaluation integrating synergy analysis, ROS/NAC rescue experiments, cytokine profiling, apoptosis- and cell cycle-associated molecular responses, immunocytochemical assessment, and 3D spheroid modeling within a TNBC framework remains limited. Therefore, the present study was designed to provide a multidimensional characterization of the biological effects associated with combined RA and DOX exposure and to further clarify its potential therapeutic relevance in breast cancer models. The obtained findings aim to provide experimental insight into the biological effects associated with combined RA and DOX exposure and to evaluate the potential therapeutic relevance of this combination in breast cancer models.

## 2. Materials and Methods

### 2.1. Cell Culture Conditions and Maintenance

In this study, the murine triple-negative breast cancer cell line 4T1 (ATCC^®^ CRL-2539™) and the immortalized human keratinocyte cell line HaCaT (Cell Lines Service (CLS), Eppelheim, Germany, catalog no. 300493) were used as cancerous and non-cancerous cellular models, respectively. The 4T1 cell line was cultured in RPMI-1640 medium (Gibco, Grand Island, NY, USA), whereas HaCaT cells were maintained in Dulbecco’s Modified Eagle Medium (DMEM; Gibco, USA). Both culture media were supplemented with 10% fetal bovine serum (FBS; Gibco, USA) and 1% penicillin–streptomycin solution (100 U/mL penicillin and 100 µg/mL streptomycin; Gibco, USA).

Cells were maintained in a humidified incubator at 37 °C under an atmosphere containing 5% CO_2_. Culture media were routinely refreshed every 2–3 days, and cells were passaged upon reaching approximately 70–80% confluence using standard trypsinization procedures with 0.25% trypsin–EDTA solution (Gibco, Grand Island, NY, USA). All experimental procedures were performed using cells between passages 3 and 8 to minimize phenotypic variability associated with prolonged culture.

Prior to treatment applications, cell morphology and growth characteristics were routinely monitored under an inverted phase-contrast microscope to confirm cellular integrity and absence of contamination. For all experiments, cells were seeded at appropriate densities according to the requirements of each assay and allowed to adhere overnight before treatment exposure.

### 2.2. Preparation of RA and DOX Treatment Solutions

RA (Sigma-Aldrich, St. Louis, MO, USA) was dissolved in dimethyl sulfoxide (DMSO; Sigma-Aldrich, USA) to prepare a 100 mM stock solution. Prior to experimental applications, serial dilutions were prepared in complete culture medium to obtain final treatment concentrations of 10, 20, 50, 100, 250, and 500 µM. Purified RA obtained from Sigma-Aldrich was selected to ensure a standardized and chemically defined experimental compound. The use of purified RA minimizes batch-to-batch variability and eliminates potential confounding effects arising from other phytochemicals that may be present in plant extracts.

DOX hydrochloride (DOX; Sigma-Aldrich, USA) was dissolved in sterile distilled water to generate the stock solution, and working concentrations of 1, 2.5, 5, 10, 25, and 50 µM were freshly prepared in complete medium immediately before use.

For combination treatment experiments (RA+DOX), simultaneous exposure protocols were established based on the half-maximal inhibitory concentration (IC_50_) values determined for each compound in preliminary cell viability analyses. The selected combination concentrations were subsequently used throughout apoptosis, mitochondrial membrane potential, ROS, cell cycle, cytokine, RT-qPCR, immunocytochemistry, NAC rescue, and 3D spheroid experiments.

The final DMSO concentration was maintained at ≤0.1% (*v*/*v*) in all experimental groups, including vehicle controls. Preliminary control experiments confirmed that this concentration did not produce detectable effects on cellular morphology or viability. Untreated control cells and vehicle-treated control groups containing equivalent DMSO concentrations were included in all experiments. Unless otherwise specified, treatment durations were applied for 24 and 48 h under standard culture conditions.

### 2.3. Evaluation of Cell Viability and Determination of IC_50_ Values

Cell viability and treatment-associated cytotoxicity were evaluated using the MTT [3-(4,5-dimethylthiazol-2-yl)-2,5-diphenyltetrazolium bromide] assay (Sigma-Aldrich, USA). Briefly, 4T1 and HaCaT cells were seeded into 96-well culture plates at a density of 5 × 10^3^ cells/well and allowed to adhere overnight under standard culture conditions.

Following incubation, cells were treated with increasing concentrations of RA, DOX, or the RA+DOX combination prepared as described above. Untreated control groups and vehicle-treated control groups containing equivalent DMSO concentrations were included in all experiments. Treatment responses were evaluated following 24 and 48 h exposure periods.

At the end of each treatment period, culture media were carefully removed, and 10 µL of MTT solution (5 mg/mL in phosphate-buffered saline (PBS)) was added to each well. Plates were subsequently incubated for 4 h at 37 °C to allow intracellular formation of purple formazan crystals by metabolically active cells. Following incubation, the supernatants were discarded, and the resulting formazan crystals were dissolved in 100 µL DMSO.

Absorbance values were measured at 570 nm using a microplate reader (BioTek Instruments, Winooski, VT, USA). Cell viability percentages were calculated relative to untreated control groups. Dose–response curves and IC_50_ values were generated using GraphPad Prism 9.0 software (GraphPad Software, San Diego, CA, USA).

For combination treatment experiments, IC_50_ values obtained from monotherapy analyses were used as operational reference points for the selection of fixed-ratio combination concentrations in subsequent mechanistic experiments. Each experimental condition was analyzed using three independent biological replicates, with each experiment performed in triplicate technical wells. All downstream experiments were performed using the IC_50_-based concentrations determined from preliminary viability assays.

### 2.4. Flow Cytometric Evaluation of Apoptotic Cell Death

Treatment-associated apoptotic cell death was analyzed using Annexin V-FITC/Propidium Iodide (PI) double staining followed by flow cytometric analysis. Briefly, 4T1 and HaCaT cells were seeded into 6-well plates and exposed to RA, DOX, or the RA+DOX combination at IC_50_-based concentrations for 48 h under standard culture conditions.

Following treatment exposure, cells were harvested using trypsin–EDTA solution, collected by centrifugation, and washed twice with cold PBS. Cell pellets were subsequently resuspended in 1× binding buffer according to the manufacturer’s protocol (BD Biosciences, San Jose, CA, USA).

For apoptotic staining, 5 µL Annexin V-FITC and 5 µL PI solution were added to each sample, followed by incubation for 15 min at room temperature in the dark. After staining, samples were immediately analyzed using a BD FACSCanto II flow cytometer (BD Biosciences, San Jose, CA, USA).

A minimum of 10,000 events was acquired for each sample. Flow cytometric data were analyzed using FlowJo v10 software (BD Biosciences, San Jose, CA, USA). Cellular populations were categorized as viable (Annexin V^−^/PI^−^), early apoptotic (Annexin V^+^/PI^−^), late apoptotic (Annexin V^+^/PI^+^), and necrotic (Annexin V^−^/PI^+^).

For quantitative comparisons, total apoptotic populations were calculated as the combined percentage of early and late apoptotic cells. Representative quadrant distributions and percentage-based population analyses were used for comparative evaluation of treatment-associated apoptotic responses.

### 2.5. Assessment of Mitochondrial Membrane Potential (ΔΨm)

Changes in mitochondrial membrane potential (ΔΨm) were evaluated using the JC-1 [5,5′,6,6′-tetrachloro-1,1′,3,3′-tetraethylbenzimidazolecarbocyanine iodide] fluorescent probe (Cayman Chemical, Ann Arbor, MI, USA). Briefly, 4T1 and HaCaT cells were seeded into 6-well plates and treated with RA, DOX, or the RA+DOX combination at IC_50_-based concentrations for 48 h.

Following treatment exposure, cells were harvested using trypsin–EDTA, washed twice with PBS, and incubated with 2 µM JC-1 dye at 37 °C for 20 min in the dark according to the manufacturer’s protocol. After staining, cells were washed twice with PBS to remove excess dye and immediately analyzed using a BD FACSCanto II flow cytometer (BD Biosciences, San Jose, CA, USA).

JC-1 accumulates within healthy mitochondria as J-aggregates, producing red fluorescence signals (FL2 channel, 590 nm), whereas mitochondrial depolarization results in conversion of JC-1 into its monomeric form, which emits green fluorescence (FL1 channel, 527 nm). Therefore, loss of ΔΨm was quantified by calculating the red/green fluorescence intensity ratio for each experimental group.

Carbonyl cyanide m-chlorophenyl hydrazone (CCCP; 10 µM), a well-established mitochondrial depolarizing agent, was used as a positive control to validate assay performance. Flow cytometric data were analyzed using FlowJo v10 software (BD Biosciences, San Jose, CA, USA), and treatment-associated alterations in mitochondrial depolarization were comparatively evaluated between experimental groups.

### 2.6. Quantification of Cytokine Secretion Profiles

To evaluate treatment-associated alterations in inflammatory signaling, the levels of pro-inflammatory and anti-inflammatory cytokines, including interleukin-1 beta (IL-1β), interleukin-6 (IL-6), interleukin-10 (IL-10), tumor necrosis factor-alpha (TNF-α), and transforming growth factor-beta (TGF-β), were quantified in culture supernatants using commercially available enzyme-linked immunosorbent assay (ELISA) kits (R&D Systems, Minneapolis, MN, USA) according to the manufacturer’s instructions.

Briefly, 4T1 and HaCaT cells were seeded into appropriate culture plates and treated with RA, DOX, or the RA+DOX combination at IC_50_-based concentrations for 48 h. Following treatment exposure, culture supernatants were collected and centrifuged at 500× *g* for 10 min to remove cellular debris. The clarified supernatants were subsequently aliquoted and stored at −80 °C until ELISA analysis.

For cytokine quantification, absorbance measurements were obtained using a microplate reader (BioTek Instruments, Winooski, VT, USA), and cytokine concentrations were calculated from corresponding standard calibration curves generated for each cytokine. Final cytokine levels were expressed as pg/mL. All measurements were performed using three independent biological replicates, and each sample was analyzed in parallel technical wells to ensure experimental reproducibility.

### 2.7. Quantitative Analysis of Apoptosis- and Cell Cycle-Related Gene Expression by RT-qPCR

Quantitative real-time polymerase chain reaction (RT-qPCR) analysis was performed to evaluate treatment-associated alterations in the expression of apoptosis- and cell cycle-related genes. Briefly, 4T1 and HaCaT cells were treated with RA, DOX, or the RA+DOX combination at IC_50_-based concentrations for 48 h under standard culture conditions.

Following treatment exposure, total RNA was isolated using TRIzol reagent (Invitrogen, Carlsbad, CA, USA) according to the manufacturer’s instructions. RNA concentration and purity were determined using a NanoDrop 2000 spectrophotometer (Thermo Fisher Scientific, Waltham, MA, USA), and only RNA samples with A260/A280 ratios between 1.8 and 2.0 were included in subsequent analyses.

Complementary DNA (cDNA) synthesis was performed using 1 µg of total RNA with the RevertAid First Strand cDNA Synthesis Kit (Thermo Fisher Scientific, Waltham, MA, USA) following the manufacturer’s protocol. Quantitative PCR amplification was subsequently carried out using SYBR Green PCR Master Mix (Applied Biosystems, Foster City, CA, USA) on a LightCycler 480 Real-Time PCR System (Roche Diagnostics, Basel, Switzerland).

Amplification reactions were performed for 40 cycles under optimized thermal cycling conditions. The analyzed apoptosis-related target genes included *BAX*, *BCL2*, *CASP3*, and *CASP9*, whereas the cell cycle-associated genes included *TP53* and *CDKN1A (p21)*. Relative gene expression levels were normalized against the housekeeping genes *GAPDH* and *ACTB (β-actin)*.

Relative fold-change values were calculated using the 2^−ΔΔCt^ method. Primer sequences used for RT-qPCR analysis were obtained from previously published literature sources and are presented in Table 1. All experiments were performed using three independent biological replicates, and each sample was analyzed in technical triplicate reactions to ensure analytical reproducibility.

### 2.8. Immunocytochemical Evaluation of Cleaved Caspase-3 Expression

Immunocytochemical analysis was performed to evaluate treatment-associated alterations in apoptotic protein expression using a diaminobenzidine (DAB)-based chromogenic staining protocol. Briefly, 4T1 and HaCaT cells were seeded onto sterile glass coverslips placed in 24-well culture plates and treated with RA, DOX, or the RA+DOX combination at IC_50_-based concentrations for 48 h.

Following treatment exposure, cells were fixed with 4% paraformaldehyde for 15 min at room temperature. Endogenous peroxidase activity was subsequently blocked by incubation with 3% hydrogen peroxide (H_2_O_2_) prepared in methanol for 10 min. For cellular permeabilization, samples were treated with 0.1% Triton X-100 prepared in PBS for 10 min.

To minimize nonspecific antibody binding, cells were incubated with 5% bovine serum albumin (BSA) blocking solution for 1 h at room temperature. Coverslips were then incubated overnight at 4 °C with a rabbit monoclonal primary antibody against cleaved caspase-3 (1:200 dilution; ab184787, Abcam, Cambridge, UK).

Following primary antibody incubation, samples were washed with PBS and subsequently incubated with horseradish peroxidase (HRP)-conjugated anti-rabbit secondary antibody (ab6721, Abcam, Cambridge, UK; 1:500 dilution) for 1 h at room temperature. Immunoreactive signals were visualized using a DAB chromogen substrate kit (Sigma-Aldrich, St. Louis, MO, USA). Cells were exposed to DAB solution for approximately 3–5 min until positive staining became visible as a brown precipitate.

After chromogenic development, nuclei were counterstained with hematoxylin for 30 s. Samples were subsequently dehydrated using increasing ethanol concentrations, cleared in xylene, and permanently mounted onto glass slides.

Immunocytochemical images were acquired using an Olympus BX53 light microscope (Olympus Corporation, Tokyo, Japan) at 20× magnification. Quantitative analysis of DAB staining intensity was performed using the color deconvolution plugin in ImageJ software (National Institutes of Health, Bethesda, MD, USA). Optical density (OD) measurements were obtained from at least five randomly selected microscopic fields for each experimental group and expressed as mean ± SD.

H-scores were calculated using the formula H-score = Σ(Pi × i), where Pi represents the percentage of cells exhibiting a given staining intensity and i represents the corresponding intensity score (0 = negative, 1 = weak, 2 = moderate, and 3 = strong). Final H-scores ranged from 0 to 300. At least five randomly selected microscopic fields were evaluated for each experimental group, and the mean H-score value was calculated and used for statistical comparisons.

### 2.9. Cell Cycle Distribution Analysis by Flow Cytometry

To further investigate whether treatment-associated cytotoxicity was accompanied by alterations in cell cycle progression, cell cycle distribution analysis was performed using PI-based flow cytometry. 4T1 cells were seeded into 6-well plates and treated with RA, DOX, or the RA+DOX combination at previously determined IC_50_ concentrations for 48 h under standard culture conditions.

Following treatment, cells were harvested using trypsin–EDTA, washed twice with cold PBS, and fixed in ice-cold 70% ethanol overnight at −20 °C. Fixed cells were subsequently centrifuged, washed with PBS to remove residual ethanol, and incubated with PI/RNase staining solution containing 50 µg/mL PI and 100 µg/mL RNase A for 30 min at room temperature in the dark.

DNA content was analyzed using a BD FACSCanto™ II flow cytometer (BD Biosciences, San Jose, CA, USA). Cell populations corresponding to SubG1, G_0_/G_1_, S, and G_2_/M phases were quantified using FlowJo software version 10.8 (BD Biosciences, San Jose, CA, USA). Cell cycle redistribution associated with treatment exposure was interpreted together with apoptosis-related findings. All experiments were performed in three independent biological replicates.

### 2.10. Determination of Intracellular ROS Levels

Intracellular ROS production was evaluated using the oxidation-sensitive fluorescent probe 2′,7′-dichlorodihydrofluorescein diacetate (DCFH-DA; Sigma-Aldrich, St. Louis, MO, USA). 4T1 and HaCaT cells were seeded into 6-well plates and treated with RA, DOX, or the RA+DOX combination at IC_50_ concentrations for 48 h under standard culture conditions.

Following treatment, cells were incubated with 10 µM DCFH-DA at 37 °C for 30 min in the dark. Cells were subsequently harvested by trypsinization, washed twice with ice-cold PBS, and immediately analyzed using a BD FACSCanto™ II flow cytometer (BD Biosciences, San Jose, CA, USA). Fluorescence signals were detected using excitation/emission settings of 488/525 nm.

Intracellular ROS levels were expressed as mean fluorescence intensity (MFI) normalized to untreated control groups and presented as fold change relative to controls. Data acquisition and analysis were performed using FlowJo software version 10.8 (BD Biosciences, San Jose, CA, USA). All experiments were conducted in three independent biological replicates.

### 2.11. NAC-Mediated Antioxidant Rescue Experiments

To functionally investigate the contribution of oxidative stress to treatment-associated cytotoxicity, antioxidant rescue experiments were performed using N-acetyl-L-cysteine (NAC; Sigma-Aldrich, St. Louis, MO, USA). 4T1 cells were pretreated with 5 mM NAC for 1 h prior to exposure to the RA+DOX combination under standard culture conditions.

Following NAC pretreatment, cells were exposed to RA+DOX treatment at IC_50_-based concentrations for 48 h. Intracellular ROS accumulation was subsequently evaluated using DCFH-DA staining and flow cytometric analysis, whereas treatment-associated viability changes were assessed using the MTT assay.

The extent to which NAC pretreatment attenuated intracellular ROS accumulation and partially restored cell viability was interpreted as a functional indicator supporting the involvement of oxidative stress in RA+DOX-associated cytotoxicity. Untreated cells, NAC-alone groups, and RA+DOX-treated groups without NAC pretreatment were included as experimental controls. All experiments were performed in triplicate.

### 2.12. Three-Dimensional (3D) Tumor Spheroid Formation and Treatment

A three-dimensional (3D) tumor spheroid model was established to evaluate treatment-associated responses under conditions partially mimicking the structural organization, diffusion limitations, and cell–cell interactions characteristic of solid tumors. 4T1 cells were maintained in RPMI-1640 medium supplemented with 10% FBS and 1% penicillin–streptomycin under standard culture conditions.

For spheroid formation, cells in the logarithmic growth phase were seeded into ultra-low-attachment round-bottom 96-well plates (Corning Inc., Corning, NY, USA) at a density of 3 × 10^3^ cells/well in a final volume of 200 µL complete culture medium. Plates were incubated at 37 °C in a humidified atmosphere containing 5% CO_2_ to allow spontaneous spheroid aggregation and maturation. Compact and morphologically uniform spheroids formed within 48–72 h and were selected for downstream analyses.

Following spheroid formation, culture medium was carefully replaced with fresh medium containing RA, DOX, or the RA+DOX combination. Treatment concentrations were selected according to IC_50_ values obtained from two-dimensional (2D) monolayer experiments and used as operational reference concentrations for 3D studies. The primary objective of the spheroid experiments was not to establish spheroid-specific IC_50_ values, but rather to evaluate whether biologically active concentrations identified in monolayer cultures retained their antitumor activity under more physiologically relevant multicellular conditions. Considering the diffusion limitations and reduced proliferation kinetics associated with multicellular spheroid structures, treatment exposure was maintained for 72 h under standard culture conditions. The 72 h exposure period was selected to allow sufficient time for treatment-associated alterations in spheroid morphology, spheroid diameter, ATP-based viability, and live/dead cell distribution to become measurable within the 3D culture system. Vehicle-treated spheroids were included as controls. Unless otherwise specified, all spheroid-based experiments were performed using at least three independent biological replicates.

#### 2.12.1. Bright-Field Imaging and Morphological Evaluation of Spheroids

Morphological alterations induced by treatment were monitored using an inverted bright-field microscope (Olympus CKX53, Olympus Corporation, Tokyo, Japan). Representative images were acquired using identical optical and exposure settings across all experimental groups to ensure comparability.

Spheroid morphology was qualitatively evaluated according to compactness, structural integrity, border regularity, and the presence of cellular dispersion or fragmentation.

#### 2.12.2. Quantitative Analysis of Spheroid Diameter

Spheroid size measurements were performed using ImageJ software (version 1.53; National Institutes of Health, Bethesda, MD, USA). For each spheroid, diameter values were calculated as the average of two perpendicular measurements obtained from the widest spheroid regions.

Quantitative analyses were conducted using spheroids derived from at least three independent experiments, and mean spheroid diameter values were expressed in micrometers (µm).

#### 2.12.3. ATP-Based Viability Analysis of 3D Tumor Spheroids

Cell viability within spheroids was determined using the CellTiter-Glo^®^ 3D Cell Viability Assay (Promega, Madison, WI, USA), according to the manufacturer’s instructions. Following treatment exposure, an equal volume of CellTiter-Glo^®^ 3D reagent was directly added to each well to facilitate complete spheroid lysis and ATP release.

Plates were incubated to allow luminescence stabilization, and luminescence intensity was measured using a microplate reader (BioTek Instruments, Winooski, VT, USA). Viability values were normalized to untreated control spheroids and expressed as percentages relative to control groups.

#### 2.12.4. Live/Dead Fluorescence Imaging and Quantification of Spheroid Cytotoxicity

Treatment-associated cytotoxicity within spheroids was further evaluated using fluorescence-based live/dead staining with Calcein-AM and Ethidium homodimer-1 (Thermo Fisher Scientific, Waltham, MA, USA). Following staining, spheroids were visualized using an inverted fluorescence microscope (Olympus CKX53, Olympus Corporation, Tokyo, Japan) equipped with appropriate excitation and emission filter sets.

Images were acquired using identical focal planes, exposure parameters, and acquisition settings across all groups without post-acquisition image enhancement or manipulation. Green fluorescence signals corresponding to viable cells and red fluorescence signals corresponding to non-viable cells were quantitatively analyzed using ImageJ software (version 1.53; National Institutes of Health).

Relative live/dead cell distributions were calculated based on fluorescence intensity measurements obtained from at least three independent biological replicates. Live/dead fluorescence patterns were interpreted together with spheroid morphology to evaluate the spatial distribution of viable and non-viable cells within 3D tumor structures.

#### 2.12.5. NAC-Mediated Antioxidant Rescue Experiments in 3D Spheroids

To further evaluate the contribution of oxidative stress to treatment-associated spheroid cytotoxicity, antioxidant rescue experiments were performed using NAC. Mature 4T1 spheroids were pretreated with NAC (5 mM; Sigma-Aldrich, St. Louis, MO, USA) for 1 h prior to exposure to the RA+DOX combination under standard culture conditions.

Following NAC pretreatment, spheroids were exposed to treatment conditions corresponding to previously determined IC_50_-based concentrations and maintained for 72 h. Untreated spheroids, NAC-alone groups, and RA+DOX-treated spheroids without NAC pretreatment were included as experimental controls.

#### 2.12.6. Quantification of Intracellular ROS Levels in 3D Spheroids

Intracellular ROS accumulation in spheroids was quantified using DCFH-DA fluorescence staining. Following treatment exposure, spheroids were enzymatically dissociated into single-cell suspensions using Accutase^®^ solution (Sigma-Aldrich, St. Louis, MO, USA) for 15 min at 37 °C. Dissociated cells were subsequently washed with PBS and incubated with 10 µM DCFH-DA for 30 min at 37 °C under light-protected conditions.

After incubation, excess fluorescent probe was removed by PBS washing, and fluorescence intensity was analyzed using a BD FACSCanto™ II flow cytometer (BD Biosciences, San Jose, CA, USA) using excitation/emission wavelengths of 488/525 nm.

Intracellular ROS levels were expressed as fold change relative to untreated control spheroids. All experiments were performed in triplicate.

#### 2.12.7. Evaluation of Spheroid Viability Following ROS Scavenging

The effect of ROS neutralization on treatment-associated cytotoxicity was further evaluated using the CellTiter-Glo^®^ 3D Cell Viability Assay (Promega, Madison, WI, USA). Following treatment exposure, an equal volume of CellTiter-Glo^®^ 3D reagent was directly added to each well to induce complete spheroid disruption and ATP release.

Luminescence intensity was measured using a microplate reader (BioTek Instruments, Winooski, VT, USA), and viability values were normalized to untreated control spheroids and expressed as percentages. The extent to which NAC pretreatment attenuated intracellular ROS accumulation and partially restored spheroid viability was interpreted as a functional indicator supporting the involvement of oxidative stress in mediating RA+DOX-associated cytotoxicity in 3D tumor spheroids.

### 2.13. Bioinformatics

#### 2.13.1. Protein–Protein Interaction (PPI) Network Analysis

Protein–protein interaction (PPI) network analysis was performed using the STRING database (Search Tool for the Retrieval of Interacting Genes/Proteins; version 12.0; https://string-db.org; accessed on 30 April 2026) to evaluate potential functional associations among the differentially expressed apoptosis- and cell cycle-related proteins identified in the experimental analyses.

The interaction network was generated using a minimum required interaction confidence score of ≥0.700, corresponding to a high-confidence interaction threshold. Interaction sources, including experimentally validated data, curated databases, co-expression profiles, and text-mining evidence, were incorporated into the analysis to improve network coverage and biological relevance.

The resulting interaction network was subsequently exported and visualized using Cytoscape software (version 3.10.1; https://cytoscape.org; accessed on 30 April 2026). Network topology parameters, including node degree and interaction connectivity, were calculated to identify proteins with potentially central interaction roles within the constructed PPI network.

#### 2.13.2. Gene Ontology (GO) Enrichment Analysis

GO enrichment analysis was performed using the “Functional Enrichment” module integrated within the STRING database (version 12.0; https://string-db.org; accessed on 30 April 2026) to evaluate the potential biological relevance of the identified target proteins and genes.

GO enrichment was analyzed separately under the categories of biological process (BP), molecular function (MF), and cellular component (CC). The entire Homo sapiens proteome was used as the reference background dataset for enrichment calculations.

Statistical significance was determined using the Benjamini–Hochberg multiple testing correction method implemented within the STRING platform. GO terms with a false discovery rate (FDR) value of <0.05 were considered significantly enriched.

For each GO category, the top 10 enriched terms showing the highest statistical significance and enrichment scores were selected and included in downstream analyses and graphical visualizations.

#### 2.13.3. Kyoto Encyclopedia of Genes and Genomes (KEGG) Pathway Enrichment Analysis

KEGG pathway enrichment analysis was performed using the “Functional Enrichment” module available within the STRING database (version 12.0; https://string-db.org; accessed on 30 April 2026). The KEGG Pathway database (https://www.kegg.jp; accessed on 30 April 2026) was used as the reference resource for pathway annotation and enrichment matching.

Statistical significance for pathway enrichment was calculated using the Benjamini–Hochberg multiple testing correction method integrated into the STRING platform. KEGG pathways with a false discovery rate (FDR) value of <0.05 were considered significantly enriched.

Enrichment outputs, including pathway names, associated gene counts, enrichment strengths, and adjusted FDR values, were exported from the STRING interface and subsequently presented using both tabular summaries and graphical visualization approaches.

### 2.14. Statistical Analysis

All experiments were performed using at least three independent biological replicates, and the obtained data are presented as mean ± standard deviation (SD). Statistical analyses were conducted using GraphPad Prism 9.0 software (GraphPad Software, San Diego, CA, USA).

Comparisons among multiple experimental groups were performed using one-way analysis of variance (ANOVA) followed by Tukey’s multiple comparison post hoc test. For pairwise comparisons between two groups, Student’s *t*-test was applied where appropriate. A *p*-value of <0.05 was considered statistically significant in all analyses.

For flow cytometric analyses, including apoptosis, mitochondrial membrane potential (ΔΨm), intracellular ROS levels, and cell cycle distribution, quantitative comparisons were performed using percentage-based population distributions and mean fluorescence intensity values obtained from FlowJo v10 software (BD Biosciences, San Jose, CA, USA).

For immunocytochemistry experiments, optical density (OD) values obtained from ImageJ analysis and H-score values derived from staining intensity and the percentage of positively stained cells were statistically compared between treatment groups. In 3D spheroid experiments, spheroid diameter measurements, ATP-based viability values, and live/dead fluorescence quantification results were comparatively analyzed using the same statistical workflow.

To evaluate the interaction profile between RA and DOX, combination index (CI) analysis was performed according to the Chou–Talalay method using CompuSyn software version 1.0 (ComboSyn Inc., Paramus, NJ, USA). CI values were interpreted as follows: CI < 1 indicated synergistic interaction, CI = 1 indicated additive interaction, and CI > 1 indicated antagonistic interaction.

For combination treatment analyses, CI calculations were used as descriptive indicators of potential pharmacological interaction patterns and were interpreted together with the experimental viability and apoptosis findings.

## 3. Results

### 3.1. Effects on Cell Viability (MTT Test)

**Figure 1:**
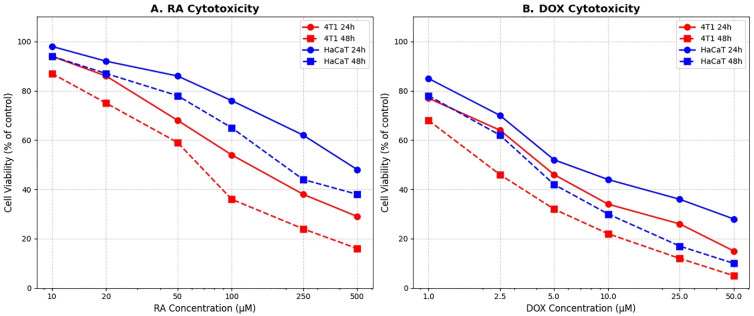
Dose-dependent cytotoxic effects of RA and DOX in 4T1 and HaCaT cells. (**A**) Cell viability following treatment with RA (10–500 µM) for 24 and 48 h. (**B**) Cell viability following treatment with DOX (1–50 µM) for 24 and 48 h. Viability was assessed by the MTT assay and expressed as a percentage of untreated controls. Red curves represent 4T1 cells and blue curves represent HaCaT cells. Solid lines indicate 24 h exposure, whereas dashed lines indicate 48 h exposure. Data are presented as mean ± SD from three independent biological replicates (*n* = 3). IC_50_ values were calculated by nonlinear dose–response analysis using GraphPad Prism 9.0.

The cytotoxic effects of RA and DOX on 4T1 murine breast cancer cells and HaCaT human keratinocyte cells were evaluated using the MTT assay following 24 and 48 h treatment exposure (Figure 1).

RA treatment induced a concentration- and time-dependent reduction in cellular viability in both cell lines (Figure 1A). In 4T1 cells, viability progressively decreased with increasing RA concentrations, and the cytotoxic effect became more pronounced following 48 h exposure compared with 24 h treatment. In contrast, HaCaT cells retained comparatively higher viability values across all tested concentrations and exposure periods.

The IC_50_ values of RA in 4T1 cells were calculated as 245.3 µM at 24 h and 178.6 µM at 48 h, whereas corresponding IC_50_ values in HaCaT cells were determined as 385.2 µM and 310.6 µM, respectively. These findings indicate that RA exerted comparatively greater cytotoxic activity against 4T1 breast cancer cells than against non-cancerous HaCaT cells (Figure 1A).

DOX treatment also produced marked concentration- and time-dependent cytotoxicity in both cellular models (Figure 1B). Compared with RA treatment, DOX induced a more pronounced reduction in cell viability, particularly at higher concentrations and prolonged exposure durations. In 4T1 cells, viability decreased to nearly 5% following 48 h treatment with 50 µM DOX. However, substantial reductions in viability were also observed in HaCaT cells, indicating lower selectivity toward cancer cells relative to RA treatment (Figure 1B).

Taken together, the obtained findings indicate that both RA and DOX significantly suppress cellular viability in 4T1 cells, whereas RA demonstrates a comparatively more selective cytotoxic profile against breast cancer cells relative to non-cancerous keratinocytes.

### 3.2. Synergistic and Selective Cytotoxic Effects of the RA+DOX Combination

**Figure 2:**
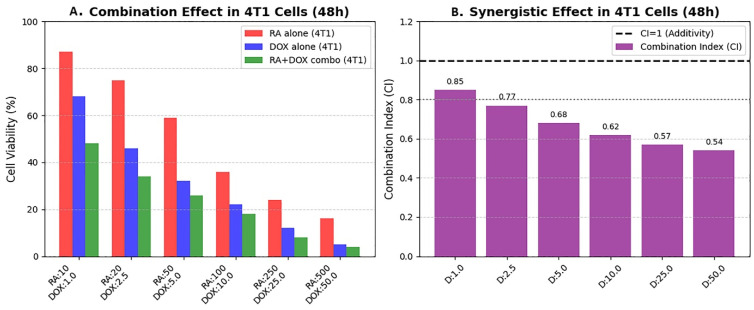
Combination effect and synergistic interaction analysis of RA and DOX in 4T1 cells following 48 h treatment. (**A**) Cell viability of 4T1 cells treated with RA alone, DOX alone, or the RA+DOX combination at corresponding concentration pairs. (**B**) Combination index (CI) analysis of the RA+DOX combination calculated using the Chou–Talalay method. The dashed black line indicates the additivity threshold (CI = 1). Cell viability was assessed using the MTT assay and expressed as a percentage of untreated controls. Data are presented as mean ± SD from three independent biological replicates (*n* = 3). CI values are shown above each bar.

**Figure 3:**
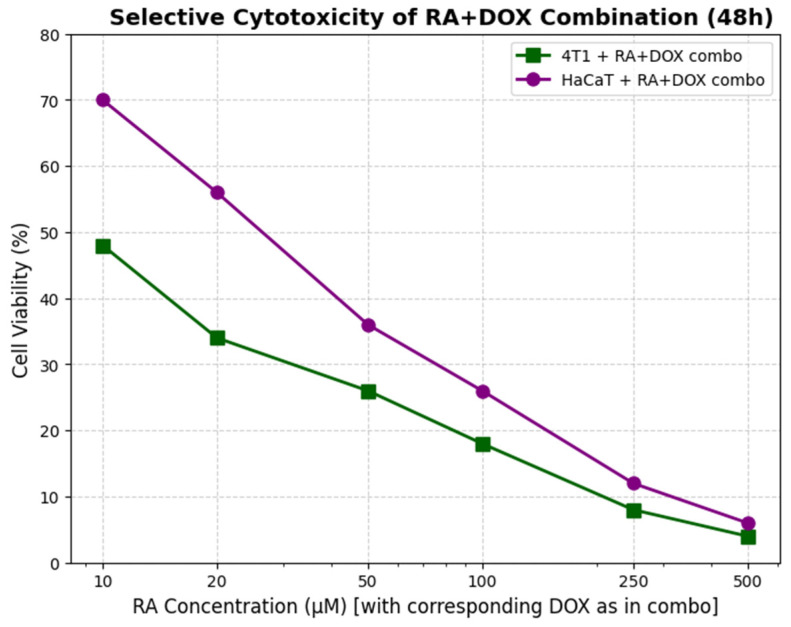
Selective cytotoxic effects of the RA+DOX combination in 4T1 and HaCaT cells following 48 h treatment. 4T1 and HaCaT cells were treated with increasing concentration pairs of RA and DOX for 48 h. Cell viability was evaluated using the MTT assay and expressed as a percentage of untreated controls. Green square symbols (■) represent 4T1 cells, whereas purple circular symbols (●) represent HaCaT cells. Data are presented as mean ± SD from three independent biological replicates (*n* = 3).

To further evaluate the combinatorial interaction between RA and DOX, 4T1 and HaCaT cells were exposed to increasing concentration pairs of RA and DOX for 48 h, and treatment-associated alterations in cellular viability were assessed using the MTT assay (Figure 2 and Figure 3).

DOX treatment alone induced marked cytotoxicity in both cell lines in a concentration- and time-dependent manner (Figure 1B). The IC_50_ values of DOX in 4T1 cells were calculated as 4.82 µM at 24 h and 2.84 µM at 48 h, whereas corresponding IC_50_ values in HaCaT cells were determined as 6.95 µM and 4.67 µM, respectively. These findings indicate that DOX exerted strong cytotoxic activity in both cancerous and non-cancerous cell lines with comparatively limited selectivity toward 4T1 cells.

Combination treatment with RA+DOX produced substantially greater reductions in 4T1 cell viability compared with either monotherapy across all tested dose pairs (Figure 2A). In particular, the combination treatment reduced viability to approximately 4% at the highest tested concentration pair (RA 500 µM + DOX 50 µM), whereas the corresponding monotherapy groups retained comparatively higher viability values.

To characterize the pharmacological interaction profile of the combination treatment, CI analysis was performed using the Chou–Talalay method (Figure 2B). All tested concentration pairs produced CI values below 1, ranging from 0.85 to 0.54, with a mean CI value of approximately 0.67, indicating synergistic interaction between RA and DOX in 4T1 cells. To further validate the interaction between RA and DOX, additional synergy analyses were performed. Detailed dose–effect data for all evaluated combinations are provided in Supplementary Table S1, while the corresponding fraction affected (Fa) values are presented in Supplementary Table S2. CI values ranged from 0.85 to 0.54 across increasing Fa levels, indicating synergistic interactions throughout the tested concentration range (Supplementary Table S3). Graphical representations of these analyses, including the Fa–CI plot and isobologram, are shown in Supplementary Figure S1.

Comparative analysis between 4T1 and HaCaT cells further demonstrated that the RA+DOX combination induced greater cytotoxicity in cancer cells relative to non-cancerous keratinocytes (Figure 3). Although cell viability progressively decreased in both cell lines with increasing treatment concentrations, HaCaT cells consistently retained higher viability values than 4T1 cells across all tested dose combinations. Because cell viability values were normalized to the corresponding untreated control group of each cell line, the observed differences primarily reflect treatment responsiveness rather than absolute growth rates. Nevertheless, direct comparisons between 4T1 and HaCaT cells should be interpreted cautiously, as these cell lines exhibit distinct intrinsic proliferation kinetics and population doubling times.

In summary, these findings indicate that the RA+DOX combination enhances cytotoxic activity in 4T1 breast cancer cells through synergistic interaction while exhibiting comparatively lower toxicity toward non-cancerous HaCaT cells.

### 3.3. Induction of Apoptotic Cell Death by RA and DOX Combination Treatment

**Figure 4:**
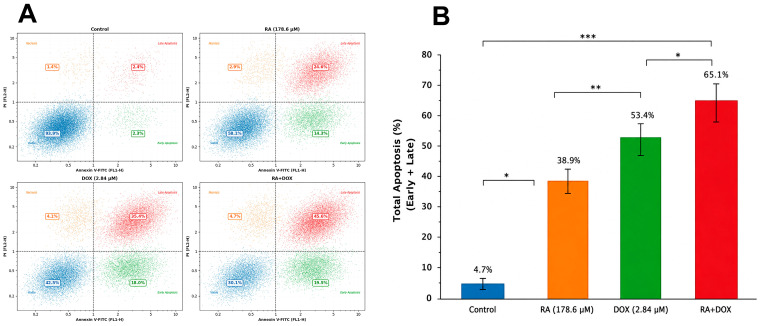
Flow cytometric evaluation of apoptotic cell populations in 4T1 cells following RA, DOX, and RA+DOX treatment. (**A**) Representative Annexin V-FITC/PI dot plots of 4T1 cells following 48 h treatment with RA (178.6 µM), DOX (2.84 µM), or the RA+DOX combination. Cell populations were analyzed according to Annexin V-FITC fluorescence (x-axis) and PI fluorescence (y-axis). Quadrants were defined as follows: Q3, viable cells (Annexin V^−^/PI^−^); Q4, early apoptotic cells (Annexin V^+^/PI^−^); Q1, late apoptotic cells (Annexin V^+^/PI^+^); and Q2, necrotic cells (Annexin V^−^/PI^+^). Percentage values indicate the proportion of cells within each gated population. (**B**) Quantitative analysis of total apoptosis, calculated as the sum of early and late apoptotic cell populations. Data are presented as mean ± SD. A minimum of 20,000 events was acquired for each experimental group. Flow cytometric analyses were performed using FlowJo v10 software (BD Biosciences, San Jose, CA, USA). * *p* < 0.05, ** *p* < 0.01, *** *p* < 0.001 versus untreated control cells.

Apoptotic cell death in 4T1 cells was evaluated using Annexin V-FITC/PI double staining followed by flow cytometric analysis after 48 h treatment exposure (Figure 4).

Untreated control cells predominantly consisted of viable populations (93.9%), whereas apoptotic cell fractions remained minimal. In the control group, early apoptotic, late apoptotic, and necrotic populations were detected at 2.3%, 2.4%, and 1.4%, respectively.

RA treatment (178.6 µM) induced a marked increase in apoptotic cell populations relative to untreated controls. The percentage of viable cells decreased to 58.1%, whereas early apoptotic and late apoptotic populations increased to 14.3% and 24.6%, respectively. Necrotic cells remained limited at 2.9%.

DOX treatment (2.84 µM) produced a more pronounced apoptotic response compared with RA monotherapy. Viable cell populations decreased to 42.5%, while early apoptotic and late apoptotic fractions increased to 18.0% and 35.4%, respectively. The necrotic population remained relatively low at 4.1%.

The strongest apoptotic response was observed in the RA+DOX combination group. Combination treatment reduced viable cell populations to 30.1% and markedly increased both early apoptotic (19.5%) and late apoptotic (45.6%) cell fractions. Necrotic cell populations remained comparatively limited (4.7%) relative to the substantial increase in apoptotic populations.

Total apoptotic cell percentages (early + late apoptosis) were calculated as 4.7% in the control group, 38.9% following RA treatment, 53.4% following DOX treatment, and 65.1% following RA+DOX combination treatment. The marked elevation observed in the combination group was primarily associated with expansion of the late apoptotic fraction.

These findings demonstrate that the RA+DOX combination substantially enhances apoptotic cell death in 4T1 cells compared with either monotherapy alone, while treatment-associated necrotic cell populations remain relatively limited.

### 3.4. Mitochondrial Membrane Potential (ΔΨm)

**Figure 5:**
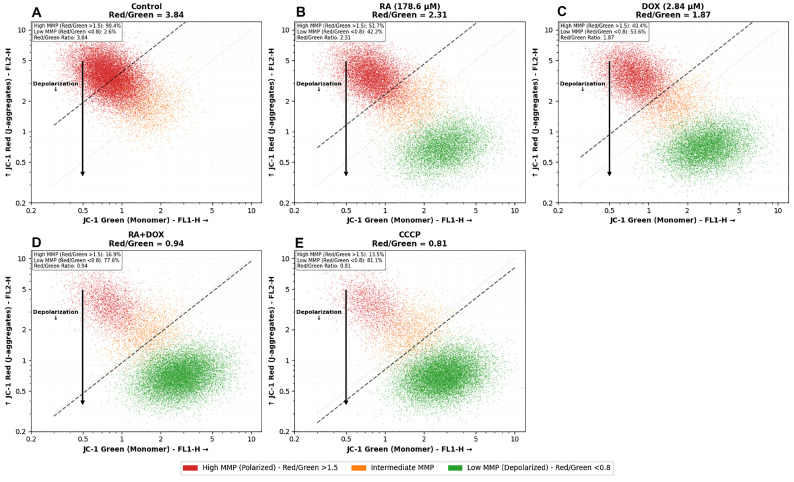
Flow cytometric JC-1 analysis of mitochondrial membrane potential (ΔΨm) in 4T1 cells following RA, DOX, and RA+DOX treatment. Representative JC-1 fluorescence distributions of 4T1 cells following 48 h treatment with (**A**) Control, (**B**) RA (178.6 µM), (**C**) DOX (2.84 µM), (**D**) RA+DOX, and (**E**) CCCP (positive control). JC-1 red fluorescence (J-aggregates; FL2-H, y-axis) represents polarized mitochondria, whereas green fluorescence (JC-1 monomers; FL1-H, x-axis) indicates depolarized mitochondria. The dashed diagonal line represents the red/green fluorescence ratio distribution for each treatment group. High-mitochondrial-membrane-potential (MMP) populations are shown in red (red/green ratio > 1.5), intermediate-MMP populations in orange, and low-MMP (depolarized) populations in green (red/green ratio < 0.8). Corresponding red/green fluorescence ratios are indicated within each panel.

Treatment-associated alterations in mitochondrial membrane potential (ΔΨm) were evaluated using JC-1 staining followed by flow cytometric analysis in 4T1 cells after 48 h of treatment exposure (Figure 5).

Untreated control cells predominantly exhibited high mitochondrial membrane polarization, as indicated by strong red JC-1 aggregate fluorescence and a high red/green fluorescence ratio (3.84 ± 0.21). Only limited mitochondrial depolarization was observed in the control group.

RA treatment (178.6 µM) induced a noticeable reduction in mitochondrial membrane polarization, resulting in a decreased red/green fluorescence ratio of 2.31 ± 0.18. Similarly, DOX treatment (2.84 µM) produced a more pronounced loss of mitochondrial membrane integrity, with the red/green ratio declining to 1.87 ± 0.14.

The most substantial mitochondrial depolarization was observed in the RA+DOX combination group. Combination treatment markedly shifted the cellular population toward increased green JC-1 monomer fluorescence, corresponding to a significant reduction in the red/green fluorescence ratio to 0.94 ± 0.09 (*p* < 0.001 vs. control). This value was comparable to that observed in the CCCP-treated positive control group (0.81 ± 0.07), indicating extensive mitochondrial membrane depolarization.

These findings indicate that the RA+DOX combination induces substantial mitochondrial dysfunction in 4T1 cells and supports the involvement of mitochondria-associated apoptotic mechanisms in treatment-associated cytotoxicity.

### 3.5. Treatment-Associated Alterations in Cytokine Profiles

**Figure 6:**
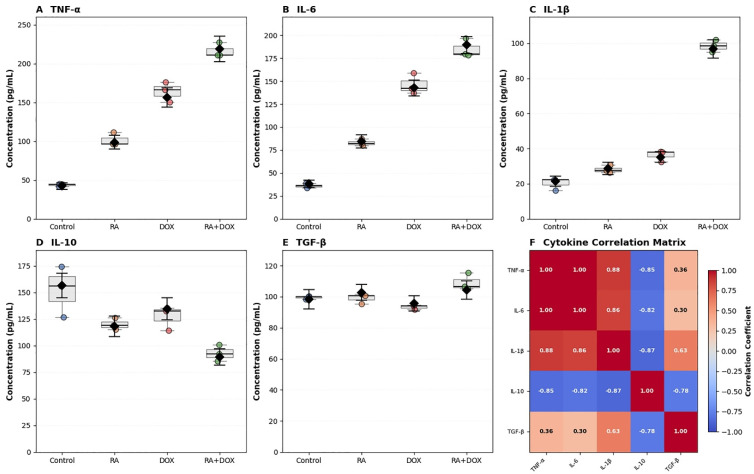
ELISA-based evaluation of cytokine secretion profiles in 4T1 cells following RA, DOX, and RA+DOX treatment. Cytokine levels were quantified in culture supernatants of 4T1 cells following 48 h treatment with RA (178.6 µM), DOX (2.84 µM), or the RA+DOX combination. (**A**) TNF-α, (**B**) IL-6, (**C**) IL-1β, (**D**) IL-10, and (**E**) TGF-β concentrations determined by ELISA. Individual colored circles represent independent biological replicates (*n* = 3). Box plots indicate median values and interquartile ranges, whereas black diamond symbols represent mean ± SD values. (**F**) Correlation matrix heatmap showing Pearson correlation coefficients among the analyzed cytokines. Warm colors indicate positive correlations, whereas cool colors indicate negative correlations.

Changes in cytokine secretion profiles following treatment exposure were evaluated in culture supernatants of 4T1 cells using ELISA analysis after 48 h incubation with RA, DOX, or the RA+DOX combination (Figure 6).

#### 3.5.1. Pro-Inflammatory Cytokines

TNF-α levels progressively increased following treatment exposure (Figure 6A). In untreated control cells, TNF-α concentration was measured as 42.3 ± 4.1 pg/mL. RA treatment increased TNF-α levels to 98.6 ± 8.7 pg/mL (*p* < 0.01 vs. control), whereas DOX treatment further elevated TNF-α secretion to 156.4 ± 12.3 pg/mL (*p* < 0.001). The highest TNF-α levels were observed in the RA+DOX combination group (218.7 ± 16.4 pg/mL), which was significantly higher than both monotherapy groups (*p* < 0.05–0.01).

IL-6 secretion demonstrated a similar increasing trend across treatment groups (Figure 6B). IL-6 concentrations increased from 38.1 ± 3.8 pg/mL in control cells to 84.3 ± 7.2 pg/mL following RA treatment (*p* < 0.01), 142.6 ± 11.8 pg/mL following DOX treatment (*p* < 0.001), and 189.4 ± 14.2 pg/mL in the RA+DOX combination group (*p* < 0.001 vs. control). Combination treatment also produced significantly higher IL-6 levels compared with RA and DOX monotherapies.

IL-1β levels exhibited comparatively moderate changes following monotherapy exposure but showed a pronounced increase in the combination group (Figure 6C). IL-1β concentrations were measured as 21.4 ± 2.9 pg/mL in control cells, 28.7 ± 3.4 pg/mL following RA treatment, and 35.2 ± 4.1 pg/mL following DOX treatment. In contrast, the RA+DOX combination markedly elevated IL-1β secretion to 96.8 ± 8.3 pg/mL (*p* < 0.001 vs. control and monotherapy groups).

#### 3.5.2. Anti-Inflammatory Cytokines

IL-10 levels demonstrated a decreasing trend following treatment exposure (Figure 6D). Control cells exhibited IL-10 concentrations of 156.7 ± 11.4 pg/mL, whereas RA treatment reduced IL-10 levels to 118.3 ± 9.6 pg/mL (*p* < 0.05). DOX treatment produced a comparatively limited reduction (134.8 ± 10.2 pg/mL), while the RA+DOX combination induced the most pronounced decrease in IL-10 secretion (89.4 ± 7.8 pg/mL; *p* < 0.001 vs. control).

In contrast, TGF-β levels remained relatively stable across all experimental groups (Figure 6E). TGF-β concentrations were measured as 98.4 ± 8.2 pg/mL in control cells, 102.7 ± 9.1 pg/mL following RA treatment, 95.6 ± 7.9 pg/mL following DOX treatment, and 104.3 ± 8.9 pg/mL following combination treatment, with no statistically significant differences detected between groups (*p* > 0.05).

Correlation matrix analysis further demonstrated strong positive correlations among the pro-inflammatory cytokines TNF-α, IL-6, and IL-1β, whereas IL-10 exhibited negative correlations with these cytokines (Figure 6F). TGF-β demonstrated comparatively weak correlation coefficients with the remaining cytokine groups.

These observations indicate that the RA+DOX combination is associated with substantial modulation of cytokine secretion profiles in 4T1 cells, particularly through increased pro-inflammatory cytokine release together with reduced IL-10 levels.

### 3.6. Treatment-Associated Alterations in Apoptosis- and Cell Cycle-Related Gene Expression

**Figure 7:**
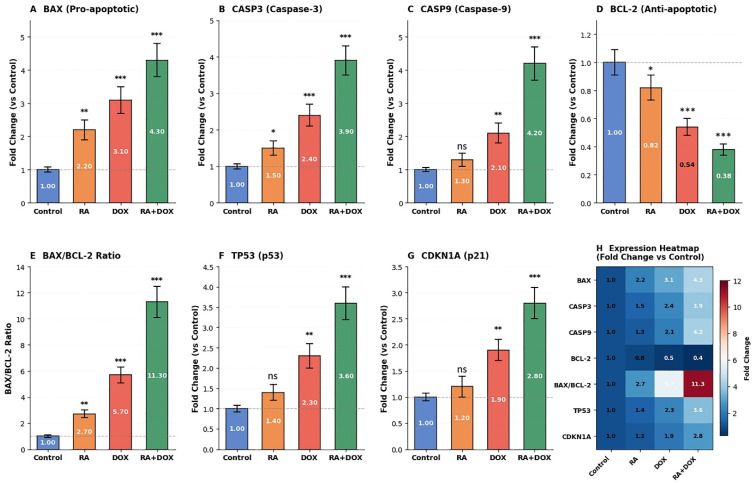
RT-qPCR analysis of apoptosis- and cell cycle-associated gene expression in 4T1 cells following RA, DOX, and RA+DOX treatment. Relative mRNA expression levels were evaluated in 4T1 cells following 48 h treatment with RA (178.6 µM), DOX (2.84 µM), or the RA+DOX combination. (**A**) *BAX*, (**B**) *CASP3*, (**C**) *CASP9*, (**D**) *BCL2*, (**E**) *BAX/BCL2* ratio, (**F**) *TP53*, and (**G**) *CDKN1A* (*p21*) expression levels presented as fold-change relative to untreated controls. (**H**) Heatmap summarizing the expression profiles of all analyzed genes across treatment groups. Data are presented as mean ± SD from three independent biological replicates (*n* = 3). Relative gene expression levels were normalized to *GAPDH* and *ACTB* reference genes and calculated using the 2^−ΔΔCt^ method. Statistical significance versus untreated controls is indicated as follows: ns, non-significant; * *p* < 0.05; ** *p* < 0.01; *** *p* < 0.001 (one-way ANOVA followed by Tukey’s post hoc test).

Expression profiles of apoptosis- and cell cycle-associated genes were evaluated in 4T1 cells using RT-qPCR following 48 h treatment exposure with RA, DOX, or the RA+DOX combination (Figure 7). Relative mRNA expression levels were normalized against *GAPDH* and *ACTB (β-actin)* reference genes.

#### 3.6.1. Pro-Apoptotic Gene Expression

Expression of the pro-apoptotic gene *BAX* progressively increased following treatment exposure (Figure 7A). Relative to untreated controls, *BAX* expression increased 2.2 ± 0.3-fold following RA treatment (*p* < 0.01), 3.1 ± 0.4-fold following DOX treatment (*p* < 0.001), and 4.3 ± 0.5-fold in the RA+DOX combination group (*p* < 0.001). Combination treatment also produced significantly higher *BAX* expression levels compared with both monotherapy groups.

Similarly, *CASP3* expression demonstrated treatment-associated upregulation across all experimental groups (Figure 7B). Relative expression levels increased 1.5 ± 0.2-fold in the RA group (*p* < 0.05), 2.4 ± 0.3-fold in the DOX group (*p* < 0.001), and 3.9 ± 0.4-fold in the RA+DOX combination group (*p* < 0.001 vs. control). The strongest increase in *CASP3* expression was observed following combination treatment.

*CASP9* expression exhibited a comparable pattern of upregulation (Figure 7C). Relative to untreated controls, *CASP9* expression increased 1.3 ± 0.2-fold following RA treatment, 2.1 ± 0.3-fold following DOX treatment (*p* < 0.01), and 4.2 ± 0.5-fold in the RA+DOX combination group (*p* < 0.001). The increase observed following combination treatment was markedly greater than that observed in either monotherapy group.

#### 3.6.2. Anti-Apoptotic Gene Expression

In contrast to the pro-apoptotic genes, expression of the anti-apoptotic gene *BCL2* progressively decreased following treatment exposure (Figure 7D). Relative *BCL2* expression decreased to 0.82 ± 0.09-fold following RA treatment (*p* < 0.05), 0.54 ± 0.06-fold following DOX treatment (*p* < 0.001), and 0.38 ± 0.04-fold following RA+DOX treatment (*p* < 0.001 vs. control). The most pronounced suppression of *BCL2* expression was observed in the combination group.

#### 3.6.3. BAX/BCL2 Expression Ratio

The *BAX/BCL2* expression ratio progressively increased across treatment groups (Figure 7E). Relative ratio values increased from 1.0 ± 0.1 in untreated control cells to 2.7 ± 0.3 following RA treatment (*p* < 0.01), 5.7 ± 0.6 following DOX treatment (*p* < 0.001), and 11.3 ± 1.2 in the RA+DOX combination group (*p* < 0.001). The markedly elevated *BAX/BCL2* ratio observed following combination treatment indicates a pronounced shift toward a pro-apoptotic transcriptional profile.

#### 3.6.4. Cell Cycle-Associated Gene Expression

Expression of the tumor suppressor gene *TP53* increased following treatment exposure (Figure 7F). Relative *TP53* expression levels increased 1.4 ± 0.2-fold following RA treatment, 2.3 ± 0.3-fold following DOX treatment (*p* < 0.01), and 3.6 ± 0.4-fold in the RA+DOX combination group (*p* < 0.001 vs. control).

Similarly, expression of *CDKN1A (p21)* progressively increased across treatment groups (Figure 7G). Relative expression values increased 1.2 ± 0.2-fold following RA treatment, 1.9 ± 0.2-fold following DOX treatment (*p* < 0.01), and 2.8 ± 0.3-fold following combination treatment (*p* < 0.001 vs. control). The strongest induction of *CDKN1A* expression was observed in the RA+DOX group.

Heatmap visualization further summarized the collective treatment-associated transcriptional alterations across all analyzed genes (Figure 7H). Combination treatment was characterized by simultaneous upregulation of pro-apoptotic and cell cycle-associated genes together with marked suppression of *BCL2* expression. These findings indicate that the RA+DOX combination substantially modulates apoptosis- and cell cycle-associated transcriptional responses in 4T1 cells, favoring a pro-apoptotic molecular profile.

### 3.7. Effects of Treatments on Cleaved Caspase-3 Expression

**Figure 8:**
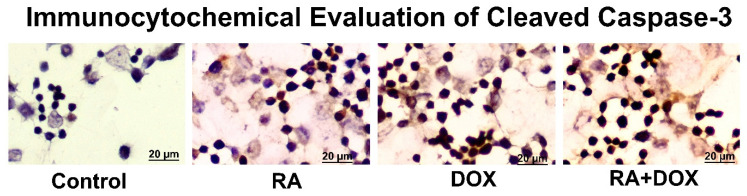
Immunocytochemical evaluation of cleaved caspase-3 expression in 4T1 cells following RA, DOX, and RA+DOX treatment. Representative immunocytochemical images of 4T1 cells following 48 h treatment with RA (178.6 µM), DOX (2.84 µM), or the RA+DOX combination. Cleaved caspase-3 expression was detected using DAB chromogen staining (brown), whereas nuclei were counterstained with hematoxylin (blue). Images were acquired using an Olympus BX53 light microscope at 20× magnification. Scale bar: 20 µm. Quantitative staining intensity values are expressed as optical density (OD) mean ± SD from three independent biological replicates (*n* = 3).

Immunocytochemical analysis using DAB-based chromogenic staining was performed to comparatively evaluate cleaved caspase-3 protein expression in 4T1 cells following treatment with RA, DOX, or the RA+DOX combination for 48 h (Figure 8).

Untreated control cells exhibited minimal cleaved caspase-3 immunoreactivity, with only weak and limited brown DAB staining observed throughout the cellular population. Quantitative optical density (OD) analysis revealed a mean staining intensity of 0.08 ± 0.01 in the control group.

RA treatment induced a noticeable increase in cleaved caspase-3 expression relative to untreated controls. Moderate cytoplasmic and partial perinuclear DAB positivity was observed in treated cells, corresponding to an OD value of 0.24 ± 0.03 (*p* < 0.01 vs. control).

DOX-treated cells demonstrated more pronounced immunoreactivity characterized by stronger and more widespread brown DAB staining, particularly in cells displaying apoptotic morphological features. Quantitative analysis demonstrated an OD value of 0.38 ± 0.04 (*p* < 0.001 vs. control).

**Figure 9:**
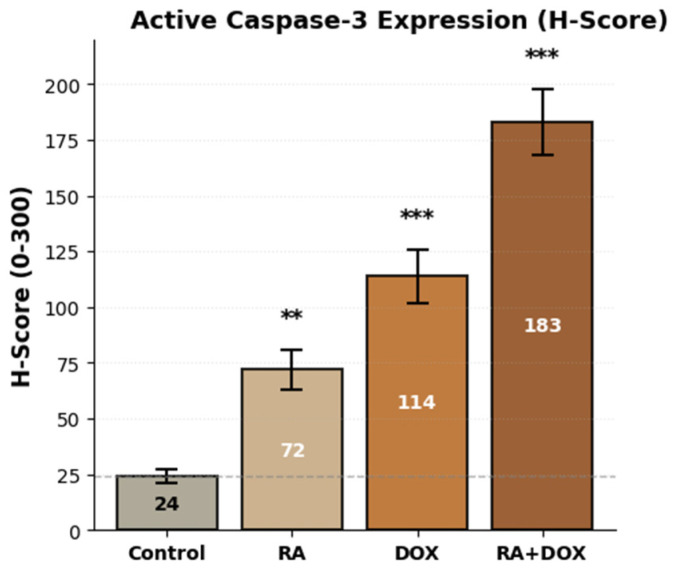
Quantitative analysis of cleaved caspase-3 immunoreactivity in 4T1 cells following RA, DOX, and RA+DOX treatment. 4T1 cells were treated with RA (178.6 µM), DOX (2.84 µM), or the RA+DOX combination for 48 h. Cleaved caspase-3 expression was detected by DAB-based immunocytochemical staining and quantitatively evaluated using H-score analysis. Bars represent mean ± SD from three independent biological replicates (*n* = 3). H-score values were calculated based on staining intensity and the percentage of positively stained cells. Statistical analysis was performed using one-way ANOVA followed by Tukey’s post hoc test. ** *p* < 0.01, *** *p* < 0.001 versus untreated control cells.

The strongest cleaved caspase-3 expression was observed in the RA+DOX combination group (Figure 9). Combination-treated cells exhibited intense and diffuse DAB positivity throughout the majority of the cellular population, together with evident apoptotic morphology. Quantitative OD analysis demonstrated a mean staining intensity of 0.61 ± 0.05 (*p* < 0.001 vs. control). These findings support the enhanced pro-apoptotic activity associated with combined RA and DOX treatment and are consistent with the flow cytometric apoptosis and RT-qPCR analyses.

### 3.8. Effects of RA+DOX Treatment on Cell Cycle Distribution

**Figure 10:**
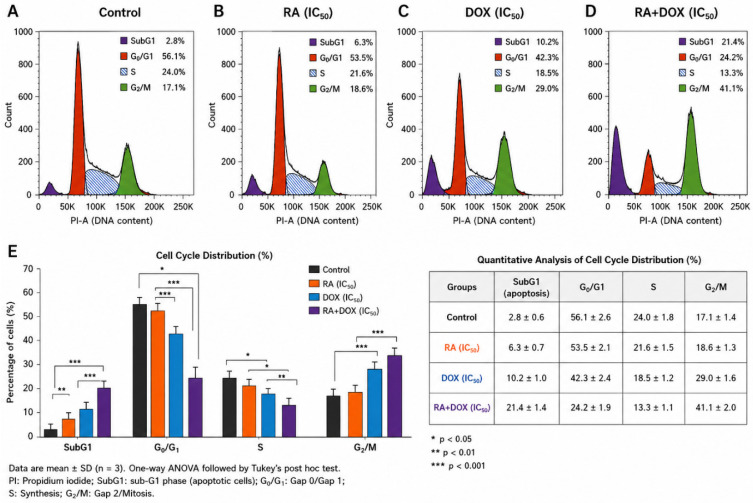
Effects of RA, DOX, and the RA+DOX combination on cell cycle distribution in 4T1 cells following 48 h treatment. 4T1 cells were treated with RA, DOX, or the RA+DOX combination at IC_50_-based concentrations for 48 h and subsequently analyzed by PI-based flow cytometry. (**A**–**D**) Representative DNA content histograms showing the distribution of cell populations within the SubG1, G_0_/G_1_, S, and G_2_/M phases. (**E**) Quantitative analysis of cell cycle phase distributions. Data are presented as mean ± SD from three independent biological replicates (*n* = 3). Statistical analysis was performed using one-way ANOVA followed by Tukey’s post hoc test. * *p* < 0.05, ** *p* < 0.01, *** *p* < 0.001 versus untreated control cells. PI, propidium iodide; SubG1, apoptotic DNA fragmentation-associated population; G_0_/G_1_, Gap 0/Gap 1 phase; S, DNA synthesis phase; G_2_/M, Gap 2/Mitosis phase.

To further investigate whether the cytotoxic and pro-apoptotic effects associated with RA+DOX treatment were accompanied by alterations in cell cycle progression, PI-based flow cytometric cell cycle analysis was performed in 4T1 cells following 48 h exposure (Figure 10).

Untreated control cells predominantly accumulated within the G_0_/G_1_ phase (56.1%), whereas the SubG1 population remained minimal (2.8%), indicating low basal apoptotic DNA fragmentation (Figure 10A,E). RA treatment induced a modest redistribution of cell cycle populations characterized by a mild increase in the SubG1 fraction (6.3%) together with partial reductions in G_0_/G_1_- and S-phase populations relative to untreated controls (Figure 10B,E).

DOX treatment produced a more pronounced alteration in cell cycle progression, characterized by marked accumulation of cells within the G_2_/M phase (29.0%), accompanied by a reduction in G_0_/G_1_-phase populations (42.3%) relative to control cells. In parallel, DOX-treated cells demonstrated a moderate increase in the SubG1 fraction (10.2%), consistent with treatment-associated apoptotic DNA fragmentation (Figure 10C,E).

Notably, the RA+DOX combination produced the most substantial redistribution of cell cycle populations. Combination treatment markedly increased both the SubG1 population (21.4%) and G_2_/M-phase accumulation (41.1%), while simultaneously reducing G_0_/G_1_- (24.2%) and S-phase (13.3%) cell populations relative to both monotherapy groups (Figure 10D,E).

Quantitative analysis confirmed that RA+DOX treatment significantly increased SubG1-associated apoptotic DNA fragmentation together with G_2_/M-phase accumulation compared with control and single-agent treatment groups (Figure 10E). Overall, these findings indicate that combined RA+DOX exposure profoundly disrupts normal cell cycle progression in 4T1 cells and promotes treatment-associated apoptotic DNA fragmentation together with G_2_/M-phase accumulation.

### 3.9. Intracellular ROS Accumulation and NAC-Mediated Antioxidant Rescue

**Figure 11:**
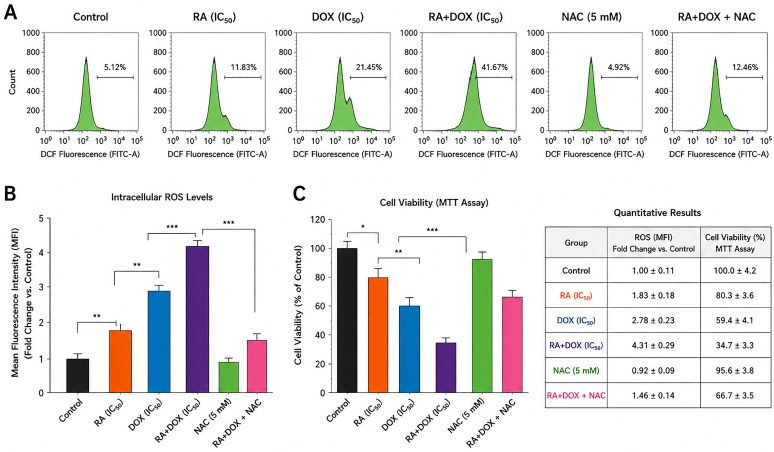
Intracellular ROS accumulation and NAC-mediated antioxidant rescue following RA and DOX treatment in 4T1 cells. 4T1 cells were treated with RA, DOX, or the RA+DOX combination at IC_50_-based concentrations for 48 h. Intracellular ROS levels were quantified using DCFH-DA fluorescence staining and flow cytometric analysis. NAC pretreatment experiments were performed to evaluate the contribution of oxidative stress to treatment-associated cytotoxicity. (**A**) Representative flow cytometry histograms showing intracellular ROS-associated DCF fluorescence intensity. (**B**) Quantitative analysis of intracellular ROS levels expressed as fold change in mean fluorescence intensity (MFI) relative to untreated controls. (**C**) Cell viability analysis following NAC-mediated antioxidant rescue. Data are presented as mean ± SD from three independent biological replicates (*n* = 3). Statistical analysis was performed using one-way ANOVA followed by Tukey’s post hoc test. * *p* < 0.05, ** *p* < 0.01, *** *p* < 0.001 versus untreated control cells. DCF, 2′,7′-dichlorodihydrofluorescein; ROS, reactive oxygen species; MFI, mean fluorescence intensity; RA, rosmarinic acid; DOX, doxorubicin; NAC, N-acetyl-L-cysteine.

To investigate whether oxidative stress contributes to the cytotoxic effects associated with RA+DOX exposure, intracellular ROS levels were quantified using DCFH-DA fluorescence analysis following 48 h treatment (Figure 11).

Representative flow cytometric histograms demonstrated progressive increases in DCF fluorescence intensity following treatment exposure, with the RA+DOX combination producing the most prominent rightward fluorescence shift among all experimental groups (Figure 7A). Untreated control cells exhibited low basal ROS levels, whereas RA treatment moderately increased intracellular ROS accumulation relative to control cells. DOX treatment induced a more pronounced elevation in ROS production compared with RA monotherapy (Figure 11A,B).

Quantitative analysis confirmed that RA treatment increased ROS fluorescence intensity to 1.83-fold relative to untreated controls, while DOX treatment increased ROS levels to 2.78-fold. Notably, the RA+DOX combination produced the highest intracellular ROS accumulation, reaching 4.31-fold relative to control cells, indicating markedly enhanced oxidative stress following combined treatment exposure (Figure 11B).

To further functionally evaluate the contribution of oxidative stress to treatment-associated cytotoxicity, antioxidant rescue experiments were performed using NAC pretreatment. NAC treatment alone did not substantially alter intracellular ROS levels relative to untreated control cells (0.92-fold). In contrast, NAC pretreatment markedly attenuated ROS accumulation in RA+DOX-treated cells, reducing fluorescence intensity from 4.31-fold to 1.46-fold relative to control levels (Figure 11A,B). This reduction in intracellular ROS was accompanied by partial restoration of cell viability. MTT analysis demonstrated that RA and DOX monotherapies reduced cell viability to 80.3% and 59.4%, respectively, whereas the RA+DOX combination induced the strongest cytotoxic effect, reducing viability to 34.7% relative to untreated controls (Figure 11C).

Importantly, NAC treatment alone maintained high cellular viability (95.6%), while NAC pretreatment significantly reversed the cytotoxic effects associated with RA+DOX exposure, partially restoring viability to 66.7% relative to combination treatment alone (Figure 11C).

When considered together, these findings support the involvement of ROS-associated oxidative stress in mediating the cytotoxic activity induced by the RA+DOX combination and demonstrate that antioxidant rescue partially attenuates combination-associated cytotoxicity in 4T1 cells. The apparently dual antioxidant/pro-oxidant behavior of RA may depend on treatment concentration, cellular context, and baseline redox status, as previously reported for several polyphenolic compounds.

### 3.10. Effects of RA+DOX Treatment on 3D Tumor Spheroid Growth and Viability

**Figure 12:**
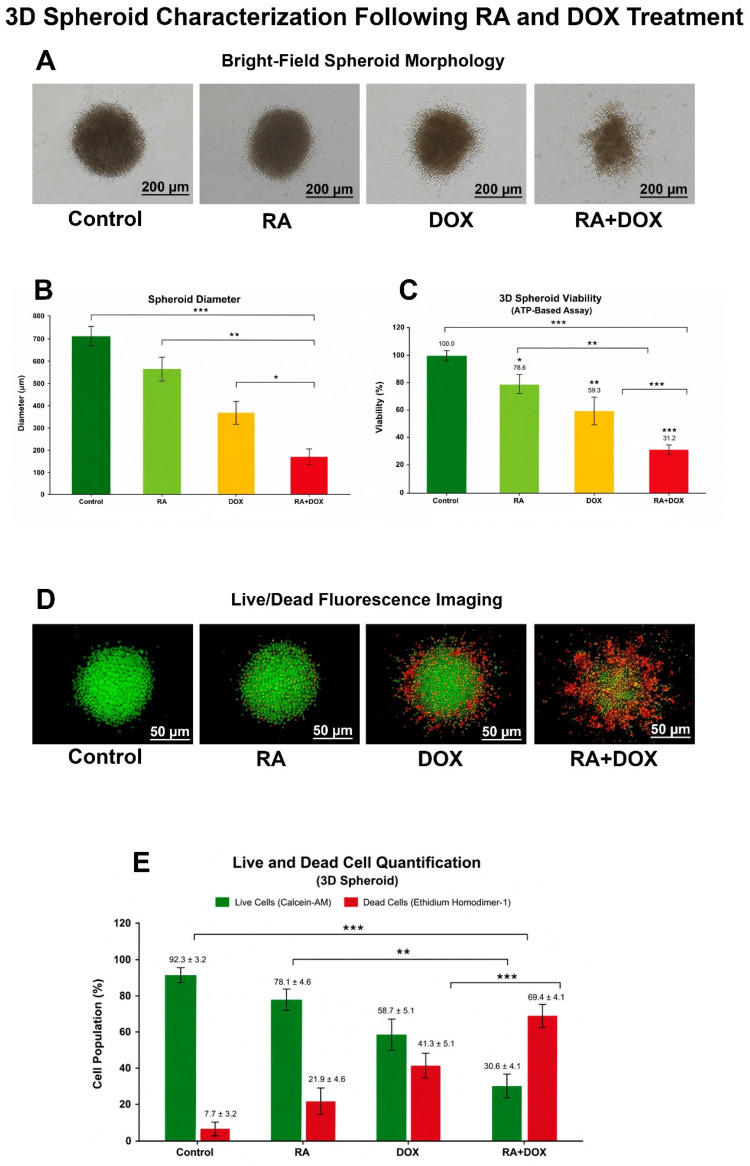
3D spheroid characterization following RA and DOX treatment. 4T1-derived multicellular tumor spheroids were treated with RA, DOX, or the RA+DOX combination at IC_50_-based concentrations for 48 h. (**A**) Representative bright-field images of untreated and treated spheroids. Scale bars = 200 µm. (**B**) Quantitative analysis of spheroid diameter. (**C**) ATP-based analysis of spheroid viability. (**D**) Representative live/dead fluorescence images of spheroids stained with Calcein-AM and Ethidium Homodimer-1. Scale bars = 200 µm. (**E**) Quantitative analysis of live and dead cell populations within spheroids. Data are presented as mean ± SD from three independent biological replicates (*n* = 3). Statistical analysis was performed using one-way ANOVA followed by Tukey’s post hoc test. * *p* < 0.05, ** *p* < 0.01, *** *p* < 0.001 versus untreated control cells.

To further evaluate treatment-associated responses under physiologically relevant tumor-like conditions, a three-dimensional (3D) 4T1 spheroid model was established and exposed to RA, DOX, or the RA+DOX combination for 48 h (Figure 12).

Representative bright-field imaging demonstrated that untreated control spheroids retained a compact spherical morphology characterized by smooth borders and dense cellular organization. RA-treated spheroids exhibited mild structural loosening together with partial reduction in spheroid compactness. In contrast, DOX treatment produced more pronounced border irregularities and peripheral structural disruption relative to control spheroids (Figure 12A).

These morphological alterations were most evident in the RA+DOX combination group, in which spheroids displayed marked architectural disintegration, irregular morphology, peripheral cellular dispersion, and substantial loss of spheroid cohesion relative to both monotherapy groups (Figure 12A).

Quantitative analysis further demonstrated that both RA and DOX monotherapies reduced spheroid diameter compared with untreated controls. Notably, combination treatment induced the greatest reduction in spheroid size, decreasing spheroid diameter from approximately 710 µm in control spheroids to nearly 170 µm following RA+DOX exposure (Figure 12B).

ATP-based viability analysis revealed that RA and DOX monotherapies moderately decreased spheroid viability to 78.6% and 59.3%, respectively, whereas the RA+DOX combination produced the strongest reduction in viable spheroid cell populations, decreasing viability to 31.2% relative to untreated controls (Figure 12C).

Consistent with these findings, live/dead fluorescence imaging demonstrated predominantly viable cell populations in untreated spheroids, as indicated by intense green fluorescence signals. RA-treated spheroids exhibited limited accumulation of red fluorescent non-viable cells, whereas DOX treatment induced more extensive dead-cell distribution together with partial disruption of spheroid architecture. The RA+DOX combination generated the highest proportion of red fluorescent non-viable cells, accompanied by severe architectural disintegration and diffuse peripheral fragmentation (Figure 12D).

Quantitative live/dead analysis further confirmed these observations. Untreated spheroids predominantly consisted of viable cells (92.3%), whereas RA and DOX monotherapies moderately increased dead-cell populations. In contrast, the RA+DOX combination produced the lowest viable-cell fraction (30.6%) together with the highest proportion of non-viable cells (69.4%) among all experimental groups (Figure 12E). Overall, these findings demonstrate that combined RA+DOX exposure exerts substantially greater suppressive effects on spheroid growth and viability than either monotherapy alone, resulting in impaired spheroid integrity, reduced viability, and increased treatment-associated cell death in 3D tumor spheroids.

### 3.11. Functional Evaluation of ROS Contribution in 3D Tumor Spheroids Using NAC Rescue

**Figure 13:**
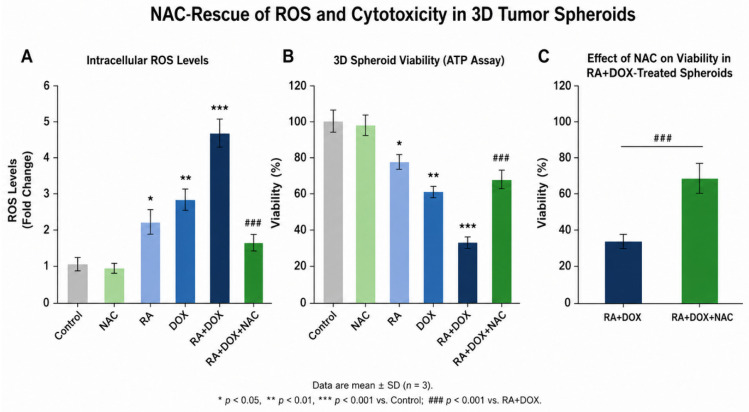
NAC-mediated modulation of ROS accumulation and cytotoxicity in 3D tumor spheroids. 4T1-derived 3D tumor spheroids were treated with RA, DOX, or the RA+DOX combination at IC_50_-based concentrations for 48 h. NAC pretreatment experiments were performed to evaluate the contribution of oxidative stress to treatment-associated cytotoxicity under 3D culture conditions. (**A**) Intracellular ROS levels quantified using the DCFDA assay. (**B**) ATP-based analysis of spheroid viability. (**C**) Direct comparison of spheroid viability between the RA+DOX and RA+DOX+NAC groups. Data are presented as mean ± SD from three independent biological replicates (*n* = 3). Statistical analysis was performed using one-way ANOVA followed by Tukey’s post hoc test. * *p* < 0.05, ** *p* < 0.01, *** *p* < 0.001 versus untreated control cells; ### *p* < 0.001 versus RA+DOX.

To further investigate whether oxidative stress contributes to treatment-associated cytotoxicity in 3D tumor spheroids, NAC-mediated antioxidant rescue experiments were performed following RA+DOX exposure (Figure 13).

Intracellular ROS analysis demonstrated that RA and DOX monotherapies moderately increased ROS production relative to untreated control spheroids. Quantitative analysis revealed that RA treatment increased ROS levels to approximately 2.2-fold, whereas DOX treatment elevated ROS accumulation to nearly 2.8-fold relative to control spheroids. Notably, the RA+DOX combination produced the highest intracellular ROS accumulation, reaching approximately 4.6-fold relative fluorescence intensity among all experimental groups (Figure 13A).

Importantly, NAC pretreatment markedly attenuated ROS elevation in RA+DOX-treated spheroids, reducing fluorescence intensity to nearly 1.6-fold relative to control levels, thereby demonstrating effective antioxidant rescue under 3D culture conditions (Figure 13A).

This reduction in oxidative stress was accompanied by partial restoration of spheroid viability. ATP-based viability analysis demonstrated that RA and DOX monotherapies reduced spheroid viability to approximately 78% and 61%, respectively, whereas the RA+DOX combination induced the strongest cytotoxic effect, reducing viability to nearly 33% relative to untreated controls (Figure 13B).

NAC treatment alone maintained high spheroid viability comparable to untreated controls. In contrast, NAC pretreatment significantly reversed the cytotoxic effects associated with RA+DOX exposure, restoring spheroid viability to approximately 68% relative to combination treatment alone (Figure 13B,C).

Direct comparison between RA+DOX and RA+DOX+NAC groups further confirmed the significant viability recovery mediated by antioxidant rescue (Figure 13C).

In combination, these findings support the involvement of ROS-associated oxidative stress in mediating the cytotoxic effects induced by RA+DOX treatment in 3D tumor spheroids and demonstrate that antioxidant intervention partially attenuates combination-associated spheroid toxicity.

### 3.12. Bioinformatic Findings

#### 3.12.1. PPI Network Analysis

**Figure 14:**
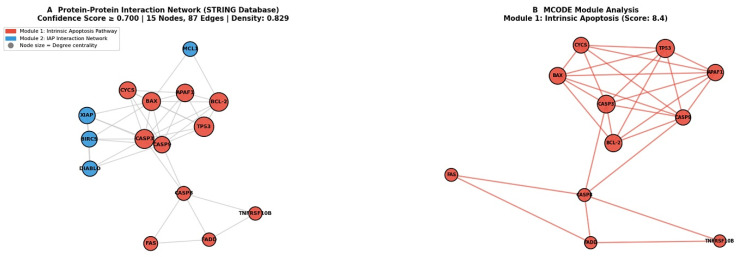
Functional annotation-based protein–protein interaction (PPI) network analysis of the predefined apoptosis- and cell cycle-related candidate-gene set. PPI network analysis was performed using the STRING database with a high-confidence interaction threshold (≥0.700), followed by network visualization and topology analysis in Cytoscape. (**A**) Global PPI network generated from the candidate genes evaluated in the experimental analyses. Node size reflects degree centrality, whereas node colors represent module assignments identified by MCODE analysis. (**B**) MCODE-derived functional interaction module associated with apoptosis-related signaling pathways, including *TP53*, *BAX*, *BCL2*, *APAF1*, *CYCS*, *CASP9*, and *CASP3*. Network topology analysis was performed using the NetworkAnalyzer and MCODE plugins in Cytoscape (v3.10.1).

The protein–protein interaction (PPI) network generated using the STRING database from the predefined apoptosis- and cell cycle-related candidate-gene set evaluated in the present study consisted of 15 nodes and 87 interaction edges at a high-confidence interaction threshold (≥0.700) (Figure 14A). Network topology analysis demonstrated a dense interaction profile with a calculated network density of 0.829 and an average clustering coefficient of 0.912, indicating strong functional interconnectivity among the analyzed proteins.

Degree centrality analysis performed using the NetworkAnalyzer plugin identified *TP53* as the major hub node within the network (degree = 14). Other highly connected proteins included *CASP3* (degree = 13), *BCL2* (degree = 12), *BAX* (degree = 11), and *APAF1* (degree = 10). In addition, network topology analysis demonstrated that *CASP3* and *CYCS* occupied important bridging positions within the interaction network, suggesting potential roles in maintaining network connectivity and apoptotic signal integration.

Module analysis performed using the MCODE plugin identified a highly interconnected intrinsic apoptosis-associated cluster with a module score of 8.4 (Figure 14B). This module included the core apoptosis-related proteins *TP53*, *BAX*, *BCL2*, *APAF1*, *CYCS*, *CASP9,* and *CASP3*, reflecting the coordinated interaction architecture of the mitochondrial apoptotic pathway.

Because the network was generated from a predefined candidate-gene panel selected on the basis of the experimental RT-qPCR findings, the resulting interactions should be interpreted as a functional annotation of the analyzed gene set rather than as an unbiased systems-level network discovery approach. These findings indicate that the apoptosis- and cell cycle-related genes evaluated in the present study are functionally interconnected within pathways associated with mitochondrial apoptosis and stress-response signaling. However, because the analysis was performed using a selected candidate-gene set rather than global transcriptomic or proteomic datasets, the results should be regarded as exploratory and hypothesis-generating.

#### 3.12.2. GO Enrichment Analysis

**Figure 15:**
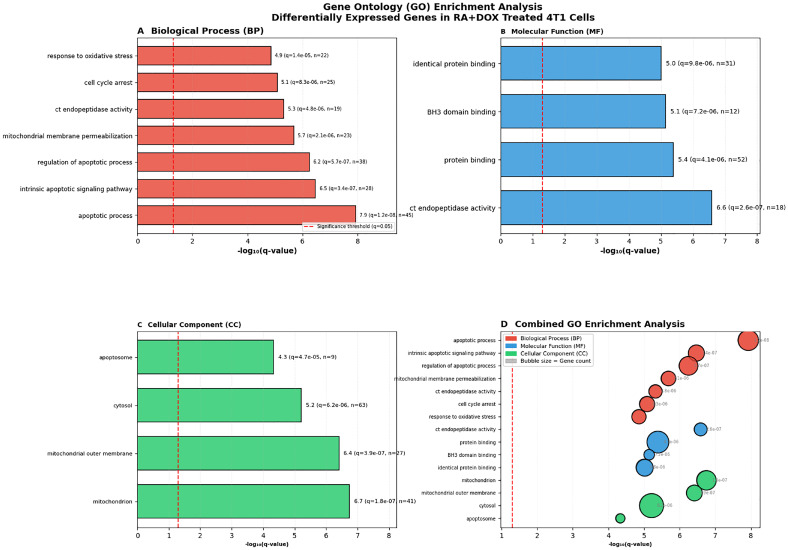
GO enrichment analysis of the predefined apoptosis- and cell cycle-related candidate-gene set following RA+DOX treatment. GO enrichment analysis was performed using apoptosis- and cell cycle-related genes evaluated following RA+DOX treatment in 4T1 cells. Significantly enriched GO terms were identified using an adjusted q-value threshold of <0.05. (**A**) Top enriched Biological Process (BP) terms. (**B**) Top enriched Molecular Function (MF) terms. (**C**) Top enriched Cellular Component (CC) terms. (**D**) Combined GO enrichment visualization summarizing significantly enriched BP, MF, and CC categories. Bubble size represents the number of genes associated with each GO term, whereas x-axis values indicate −log_10_(q-value). The dashed red line denotes the significance threshold (q = 0.05). GO enrichment analysis was performed using the STRING Functional Enrichment module with Benjamini–Hochberg multiple testing correction.

GO enrichment analysis was performed to functionally characterize the biological pathways and molecular processes associated with the differentially expressed apoptosis-related genes identified following RA+DOX treatment (Figure 15).

Within the Biological Process (BP) category, the most significantly enriched terms included apoptotic process (GO:0006915; q = 1.2 × 10^−8^), intrinsic apoptotic signaling pathway (GO:0097193; q = 3.4 × 10^−7^), regulation of apoptotic process (GO:0042981; q = 5.7 × 10^−7^), mitochondrial membrane permeabilization (GO:0051881; q = 2.1 × 10^−6^), cysteine-type endopeptidase activity involved in apoptotic process (GO:0097153; q = 4.8 × 10^−6^), cell cycle arrest (GO:0007050; q = 8.3 × 10^−6^), and response to oxidative stress (GO:0006979; q = 1.4 × 10^−5^) (Figure 15A).

Analysis of the Molecular Function (MF) category demonstrated significant enrichment of cysteine-type endopeptidase activity (GO:0004197; q = 2.6 × 10^−7^), protein binding (GO:0005515; q = 4.1 × 10^−6^), BH3 domain binding (GO:0051434; q = 7.2 × 10^−6^), and identical protein binding (GO:0042802; q = 9.8 × 10^−6^) (Figure 15B).

Within the Cellular Component (CC) category, the mitochondrion (GO:0005739; q = 1.8 × 10^−7^), mitochondrial outer membrane (GO:0005741; q = 3.9 × 10^−7^), cytosol (GO:0005829; q = 6.2 × 10^−6^), and apoptosome (GO:0043293; q = 4.7 × 10^−5^) were identified among the most significantly enriched cellular structures (Figure 15C).

Combined GO enrichment visualization further demonstrated clustering of apoptosis-associated BP, MF, and CC categories within mitochondria-associated apoptotic signaling pathways (Figure 15D).

As a whole, these findings provide functional annotation of the predefined apoptosis- and cell cycle-related candidate-gene set evaluated in the present study. The enrichment of apoptosis-associated processes, mitochondrial membrane permeabilization, oxidative stress responses, and cell cycle arrest is consistent with the experimental observations obtained from apoptosis, mitochondrial membrane potential, ROS, and cell cycle analyses. In the context of TNBC, these biological processes have been implicated in the regulation of treatment-induced apoptosis, suppression of cellular proliferation, and modulation of therapeutic responsiveness. However, because the enrichment analysis was generated from a selected candidate-gene panel rather than unbiased transcriptomic or proteomic datasets, these findings should be interpreted as exploratory and hypothesis-generating rather than definitive mechanistic evidence.

#### 3.12.3. KEGG Pathway Enrichment Analysis

**Figure 16:**
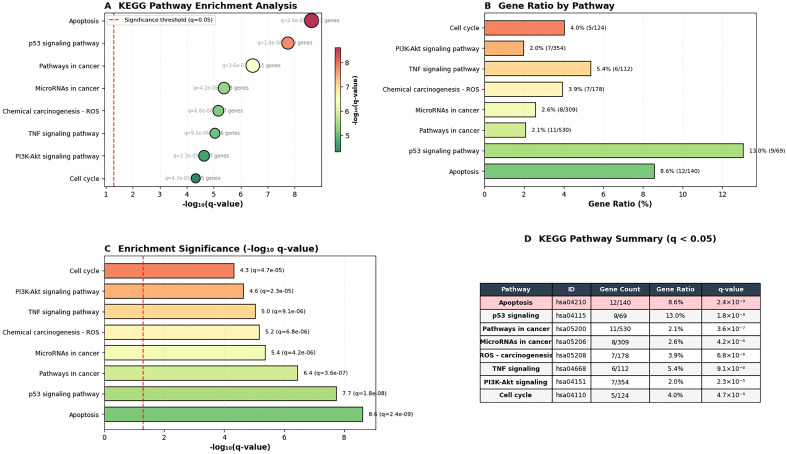
KEGG pathway enrichment analysis of the predefined apoptosis- and cell cycle-related candidate-gene set following RA+DOX treatment. KEGG pathway enrichment analysis was performed using the candidate genes evaluated in RA+DOX-treated 4T1 cells. (**A**) Bubble plot showing enriched KEGG pathways. Bubble size represents the number of genes associated with each pathway, whereas color intensity reflects enrichment significance expressed as −log_10_(q-value). (**B**) Bar graph presenting gene ratios for significantly enriched pathways. (**C**) Enrichment significance visualization showing −log_10_(q-value) values for significantly enriched KEGG pathways. The dashed red line indicates the significance threshold (q = 0.05). (**D**) Summary table presenting enriched KEGG pathways together with pathway IDs, gene counts, gene ratios, and adjusted q-values. KEGG enrichment analysis was performed using the STRING Functional Enrichment module with Benjamini–Hochberg multiple testing correction.

KEGG pathway enrichment analysis identified multiple significantly enriched signaling pathways associated with apoptosis, oxidative stress response, and cell cycle regulation following RA+DOX treatment in 4T1 cells (Figure 16).

Among the enriched pathways, the apoptosis pathway (hsa04210) demonstrated the strongest enrichment significance, with 12 of 140 pathway-associated genes represented (gene ratio: 8.6%; q = 2.4 × 10^−9^) (Figure 16A–D). The p53 signaling pathway (hsa04115) also demonstrated substantial enrichment, involving 9 of 69 genes (gene ratio: 13.0%; q = 1.8 × 10^−8^), representing the highest gene ratio among all identified pathways (Figure 16B).

Additional significantly enriched pathways included pathways in cancer (hsa05200; 11/530 genes; q = 3.6 × 10^−7^), microRNAs in cancer (hsa05206; 8/309 genes; q = 4.2 × 10^−6^), chemical carcinogenesis-ROS (hsa05208; 7/178 genes; q = 6.8 × 10^−6^), TNF signaling pathway (hsa04668; 6/112 genes; q = 9.1 × 10^−6^), PI3K–Akt signaling pathway (hsa04151; 7/354 genes; q = 2.3 × 10^−5^), and cell cycle pathway (hsa04110; 5/124 genes; q = 4.7 × 10^−5^).

Visualization of pathway enrichment significance further demonstrated that apoptosis- and p53-associated signaling pathways represented the most strongly enriched functional categories identified in the dataset (Figure 16C).

Collectively, these findings provide functional annotation of the predefined apoptosis- and cell cycle-related candidate-gene set evaluated in the present study. The enrichment of apoptosis, p53 signaling, TNF signaling, PI3K–Akt signaling, oxidative stress-associated pathways, and cell cycle regulation is consistent with the experimental observations obtained from apoptosis, ROS, mitochondrial membrane potential, and cell cycle analyses. In the context of TNBC, these pathways have been implicated in the regulation of tumor cell survival, treatment-induced apoptosis, stress adaptation, and therapeutic responsiveness. However, because the enrichment analysis was generated from a selected candidate-gene panel rather than unbiased transcriptomic or proteomic datasets, these findings should be interpreted as exploratory and hypothesis-generating rather than definitive mechanistic evidence.

## 4. Discussion

In this study, the cytotoxic, pro-apoptotic, oxidative stress-associated, and immunomodulatory effects of the combination of RA, a naturally occurring polyphenolic compound, with the conventional chemotherapeutic agent DOX were comprehensively investigated in the 4T1 murine breast cancer model using multidimensional experimental approaches. The findings demonstrated that the RA+DOX combination induced significantly enhanced cytotoxicity, promoted apoptotic cell death, disrupted mitochondrial membrane integrity, altered oxidative stress responses, modulated cytokine secretion patterns, induced cell cycle redistribution, and markedly suppressed 3D spheroid growth and viability compared with either monotherapy alone. These findings cumulatively support the potential of RA as a chemosensitizing adjunct in DOX-based breast cancer therapy [18]. MTT assay results demonstrated that both RA and DOX induced concentration- and time-dependent reductions in cellular viability in 4T1 cells. However, the most notable finding was that the RA+DOX combination consistently produced substantially greater cytotoxicity than either agent administered alone, while CI (CI < 1) analysis confirmed synergistic interaction between the two compounds. These findings are consistent with previous studies performed in ovarian adenocarcinoma and keratinocyte models, in which RA enhanced DOX sensitivity and suppressed cancer cell proliferation [10,11,12,19]. Several mechanisms may contribute to this synergistic interaction. Previous reports have suggested that RA suppresses P-glycoprotein (P-gp)-associated multidrug resistance through downregulation of *MDR1* transcription, thereby increasing intracellular accumulation of DOX and potentiating its cytotoxic activity [3,6]. In the present study, the comparatively preserved viability observed in HaCaT cells relative to 4T1 cells suggests that RA may contribute to a relatively selective cytotoxic profile favoring cancer cells. This observation may partly be associated with the antioxidant capacity of RA, which has previously been suggested to alleviate oxidative stress in healthy cells while facilitating excessive ROS accumulation in malignant cells under treatment-associated stress conditions [13,20,21]. Although rosmarinic acid has been extensively investigated as a bioactive polyphenol with anticancer potential, studies specifically evaluating its combination with doxorubicin or other anthracycline-based therapies remain relatively limited. Previous studies performed in OVCAR3 ovarian adenocarcinoma cells demonstrated that RA enhanced the antiproliferative activity of doxorubicin and modulated multiple apoptosis-associated pathways, including EGFR-/BCL2-related signaling and FOXP3-associated regulatory mechanisms, while promoting caspase-dependent apoptotic responses [11,12]. In addition, recent evidence obtained using MDA-MB-231 breast cancer cells suggested that RA may improve the therapeutic profile of doxorubicin by reducing treatment-associated cardiotoxicity without compromising its anticancer efficacy [22]. Collectively, these findings support the rationale for combining RA with anthracycline-based chemotherapy. The present findings are generally consistent with these observations while extending the available evidence to a multidimensional 4T1 triple-negative breast cancer model incorporating quantitative synergy analysis, ROS/NAC rescue experiments, cytokine profiling, immunocytochemical validation, cell cycle assessment, and three-dimensional spheroid analyses.

Flow cytometric apoptosis analysis and mitochondrial membrane potential (ΔΨm) measurements demonstrated that the RA+DOX combination strongly promoted apoptosis-associated cellular alterations in 4T1 cells. Combination treatment produced the highest apoptotic cell fraction together with the most pronounced mitochondrial depolarization, as evidenced by a substantial reduction in the JC-1 red/green fluorescence ratio. Mitochondrial membrane destabilization is known to facilitate cytochrome c release into the cytosol, apoptosome formation through APAF1 activation, and subsequent activation of the caspase-9/caspase-3 cascade [23,24,25]. Consistent with these mechanisms, RT-qPCR analyses demonstrated marked upregulation of *BAX*, *CASP3*, and *CASP9* expression together with suppression of *BCL2* expression following combination treatment. Previous studies have similarly shown that RA shifts the Bax/Bcl-2 balance toward a pro-apoptotic state in various cancer models [13]. The present findings therefore support the possibility that RA enhances the susceptibility of 4T1 cells to DOX-associated mitochondrial dysfunction and apoptosis.

The newly incorporated ROS and NAC rescue experiments further demonstrated that oxidative stress modulation may contribute to the biological activity associated with the RA+DOX combination. Intracellular ROS accumulation was markedly increased following combination treatment in both monolayer cultures and 3D spheroids, whereas NAC pretreatment substantially attenuated ROS levels and partially restored cellular viability. These observations support the involvement of ROS-associated oxidative stress in treatment-related cytotoxicity. Nevertheless, ROS modulation alone cannot fully explain the observed biological effects, and the present findings should be interpreted cautiously as evidence of a multifactorial response involving mitochondrial dysfunction, apoptosis signaling, transcriptional alterations, and oxidative stress-associated mechanisms rather than a single dominant pathway [13,20,21].

Cell cycle analyses additionally demonstrated that the RA+DOX combination induced marked redistribution of cell cycle populations characterized by increased SubG1 accumulation together with pronounced G_2_/M-phase arrest. These findings were accompanied by elevated *TP53* and *CDKN1A (p21)* expression levels, both of which are associated with DNA damage responses and cell cycle checkpoint regulation. Increased *TP53* expression may contribute to apoptosis induction and cell cycle arrest through transcriptional activation of downstream target genes, whereas *CDKN1A* upregulation may facilitate inhibition of cyclin-dependent kinase activity at G_1_/S and G_2_/M checkpoints [26]. These findings suggest that the RA+DOX combination suppresses cellular proliferation through coordinated induction of apoptosis and disruption of cell cycle progression.

Cytokine profiling analyses demonstrated that the RA+DOX combination substantially altered inflammatory mediator secretion patterns in 4T1 cells. Combination treatment produced marked increases in TNF-α, IL-6, and IL-1β levels together with decreased IL-10 secretion. Although these findings may initially appear paradoxical, they may reflect treatment-associated inflammatory responses secondary to extensive tumor cell injury. DOX has previously been reported to induce immunogenic cell death-associated signaling in certain experimental settings [27]. Therefore, the observed cytokine alterations may indicate that the RA+DOX combination modulates treatment-associated inflammatory pathways in addition to its direct cytotoxic effects. Reduced IL-10 levels may also suggest partial suppression of anti-inflammatory signaling mechanisms associated with tumor immune evasion [28]. In contrast, the absence of substantial changes in TGF-β levels suggests that the combination treatment may preferentially influence intracellular stress-associated signaling rather than broadly affecting all cytokine pathways within the tumor microenvironment [29]. Nevertheless, because the current study was performed exclusively in vitro, interpretation of these cytokine findings should remain cautious and descriptive.

A comprehensive evaluation of RT-qPCR and bioinformatic analyses further demonstrated that the RA+DOX combination induced coordinated transcriptional alterations associated with apoptosis, oxidative stress response, and cell cycle regulation. Significant increases in *BAX*, *CASP3*, *CASP9*, *TP53*, and *CDKN1A* expression together with suppression of *BCL2* indicated a pronounced pro-apoptotic transcriptional profile [26,30,31]. In parallel, protein–protein interaction (PPI), GO enrichment, and KEGG pathway analyses demonstrated enrichment of apoptosis-associated, mitochondrial, oxidative stress-related, and p53-associated signaling pathways. The identified enrichment of apoptosis, intrinsic apoptotic signaling, mitochondrial membrane permeabilization, TNF signaling, PI3K-Akt signaling, and p53-associated pathways supports the molecular findings obtained experimentally. The transcriptional alterations observed in the present study are consistent with previous reports describing the pro-apoptotic activity of rosmarinic acid in different cancer models. Liu et al. demonstrated that RA-induced apoptosis in human glioma cells was accompanied by downregulation of Bcl-2 together with increased Bax and cleaved caspase-3 expression, supporting the ability of RA to shift the cellular balance toward apoptosis [32]. Similarly, Mahmoud et al. reported that RA enhanced apoptotic responses in a breast cancer model through modulation of the p53/caspase-3 axis and suppression of the Bcl-2/Bax ratio, further supporting the involvement of apoptosis-related signaling pathways in RA-mediated anticancer activity [33]. In addition, Messeha et al. demonstrated that RA induced apoptosis and cell cycle arrest in triple-negative breast cancer cells while significantly altering the expression of multiple apoptosis- and stress-response-related genes [34]. Collectively, these studies provide biological support for the increased *BAX*, *CASP3*, *CASP9*, *TP53*, and *CDKN1A* expression together with reduced *BCL2* expression observed in the present study. However, these bioinformatic analyses should be interpreted as exploratory and hypothesis-generating rather than definitive mechanistic proof, since they were derived from selected candidate-gene datasets rather than global transcriptomic sequencing approaches.

The incorporation of 3D spheroid experiments further strengthened the physiological relevance of the present study. Compared with conventional monolayer cultures, the RA+DOX combination induced marked disruption of spheroid morphology, substantial reductions in spheroid diameter, decreased ATP-based viability, and increased dead-cell accumulation within 3D tumor spheroids. Moreover, NAC rescue experiments performed in spheroids partially restored viability and attenuated ROS accumulation, supporting consistency between 2D and 3D experimental findings. Since spheroid systems more closely resemble in vivo tumor architecture and diffusion-limited therapeutic responses, these observations provide additional support for the antitumor potential of the RA+DOX combination under more physiologically relevant conditions.

Immunocytochemical analyses additionally supported the molecular findings at the protein level. Combination treatment induced the strongest cleaved caspase-3 immunoreactivity together with marked increases in H-score values relative to monotherapy groups. These observations were consistent with the apoptosis and RT-qPCR findings and further support activation of apoptosis-associated pathways following RA+DOX exposure. Similar immunocytochemical alterations have previously been reported in OVCAR3 ovarian cancer models treated with RA and DOX combinations [10].

The present study possesses several important strengths, including the use of multidimensional analytical approaches, evaluation of both cancerous and non-cancerous cell models, incorporation of ROS/NAC rescue experiments, inclusion of cell cycle analyses, validation of findings in 3D spheroid systems, and quantitative confirmation of synergistic interaction using the Chou–Talalay method. Nevertheless, several limitations should also be considered. First, all experiments were performed exclusively in vitro; therefore, the findings cannot fully reproduce the complexity of the in vivo tumor microenvironment, pharmacokinetic behavior, immune interactions, stromal signaling, vascularization, or systemic toxicity profiles. Second, only a single murine TNBC cell line and one non-cancerous keratinocyte cell line were evaluated, limiting the generalizability of the findings across heterogeneous human breast cancer subtypes. Although HaCaT cells are widely used for preliminary safety and selectivity assessments in anticancer studies, they do not fully recapitulate the biological characteristics of normal mammary epithelial cells. Therefore, the observed selectivity profile of the RA+DOX combination should be interpreted with caution. Future studies incorporating non-tumorigenic mammary epithelial models, such as MCF-10A cells, will be important to further validate the selectivity and safety profile of this combination strategy. Third, although immunocytochemical analyses provided limited protein-level validation, comprehensive protein validation techniques such as Western blotting were not performed for the majority of apoptosis- and cell cycle-associated markers. Furthermore, although significant treatment-associated alterations were observed at the mRNA level for apoptosis- and cell cycle-related genes, these findings should be interpreted as preliminary transcriptional observations. Because comprehensive protein-level validation was not performed for most targets, the relationship between mRNA expression and functional protein abundance remains to be confirmed. Future studies incorporating Western blotting or other quantitative protein-based approaches will be necessary to validate the biological relevance of these transcriptional alterations. Fourth, ROS analyses were primarily based on DCFH-DA fluorescence and NAC rescue experiments, both of which possess recognized methodological limitations regarding specificity and mechanistic interpretation. Fifth, cytokine profiling was restricted to a selected panel of soluble mediators and therefore cannot fully characterize treatment-associated immune responses or immunogenic cell death mechanisms. Sixth, although 3D spheroid systems provide improved physiological relevance compared with monolayer cultures, they still lack stromal, vascular, and immune microenvironment components present in vivo. In addition, spheroid-specific dose–response analyses and 3D IC_50_ determinations were not performed. Instead, spheroid experiments were conducted using biologically active concentrations identified in monolayer cultures to evaluate whether the observed treatment effects were maintained under multicellular conditions. Future studies incorporating dedicated 3D dose–response and time-course analyses would provide a more comprehensive characterization of treatment efficacy in spheroid models. Seventh, the bioinformatic analyses were exploratory in nature and generated from selected candidate-gene datasets rather than unbiased transcriptomic or proteomic sequencing approaches. Eighth, the present study evaluated purified rosmarinic acid rather than RA-rich plant extracts. Because natural extracts contain multiple bioactive phytochemicals that may exert additive, synergistic, or antagonistic biological effects, the findings reported here specifically reflect the activity of purified RA under controlled experimental conditions. Future studies comparing purified RA with RA-rich natural extracts may provide additional insight into the contribution of other phytochemical constituents to the observed anticancer effects and further improve the translational relevance of this therapeutic strategy. Finally, the pharmacological bioavailability and metabolic stability of RA remain important translational challenges, as polyphenolic compounds frequently exhibit limited systemic bioavailability following conventional administration routes. Recent studies investigating nanoparticle- and liposome-based delivery systems may help overcome these limitations and improve therapeutic applicability. Additionally, previous investigations demonstrating the protective effects of RA against DOX-associated cardiotoxicity further support the translational relevance of this combination strategy [35,36,37].

Taken together, the present findings demonstrate that the RA+DOX combination exerts multifaceted antitumor effects in 4T1 breast cancer cells through coordinated modulation of apoptosis-associated, oxidative stress-related, mitochondrial, inflammatory, and cell cycle-associated responses. Future investigations incorporating in vivo TNBC models, broader protein-level validation, mechanistic pathway analyses, immune microenvironment characterization, and optimized RA delivery systems will be necessary to further clarify the translational potential of this combination strategy in breast cancer therapy.

## 5. Conclusions

The present study demonstrated that the combination of RA and DOX exerts enhanced antitumor activity in 4T1 breast cancer cells compared with either monotherapy alone. Combination treatment induced marked reductions in cellular viability, increased apoptotic cell death, disrupted mitochondrial membrane potential, altered oxidative stress responses, promoted cell cycle redistribution, and suppressed 3D spheroid growth and viability under tumor-like culture conditions.

The observed biological effects were accompanied by increased expression of apoptosis-associated genes, including *BAX*, *CASP3*, *CASP9*, *TP53*, and *CDKN1A*, together with suppression of the anti-apoptotic gene *BCL2*. In parallel, cleaved caspase-3 immunoreactivity was markedly increased following combination treatment, supporting apoptosis-associated activation at both the transcriptional level and through increased cleaved caspase-3 immunoreactivity. ROS/NAC rescue experiments further suggested that oxidative stress-associated mechanisms may contribute to the cytotoxic activity of the RA+DOX combination, although these effects likely occur within a broader multifactorial response involving mitochondrial dysfunction, apoptosis-associated pathways, and cell cycle regulation.

Bioinformatic enrichment analyses additionally demonstrated a significant association of the altered molecular profile with apoptosis-related, mitochondrial, oxidative stress-associated, and p53-regulated signaling pathways. Furthermore, 3D spheroid experiments confirmed that the RA+DOX combination remained biologically active under diffusion-limited tumor-like conditions and produced substantial disruption of spheroid integrity together with reduced spheroid viability.

Considered together, these findings suggest that RA may enhance the antitumor activity of DOX through coordinated modulation of apoptosis-associated, oxidative stress-related, mitochondrial, and cell cycle-associated responses in breast cancer cells. However, because the present study was performed exclusively in vitro, the findings should be interpreted cautiously and considered preliminary. Additional investigations using human-derived TNBC models, broader protein-level validation, and in vivo experimental systems will be necessary to further clarify the translational and therapeutic potential of this combination strategy in breast cancer treatment.

## Figures and Tables

**Table 1:** Primer sequences used for quantitative real-time PCR analysis. Forward and reverse primer sequences used for the amplification of apoptosis- and cell cycle-associated target genes and housekeeping reference genes in 4T1 cells. Relative gene expression levels were normalized against ACTB (β-actin) and GAPDH reference genes and calculated using the 2^−ΔΔCt^ method.

Gene	Forward Primer (5′→3′)	Reverse Primer (5′→3′)
*CASP3*	GGAAGCGAATCAATGGACTCTGG	GCATCGACATCTGTACCAGACC
*CASP9*	GTTTGAGGACCTTCGACCAGCT	CAACGTACCAGGAGCCACTCTT
*BAX*	TCAGGATGCGTCCACCAAGAAG	TGTGTCCACGGCGGCAATCATC
*BCL2*	ATCGCCCTGTGGATGACTGAGT	GCCAGGAGAAATCAAACAGAGGC
*TP53*	CCTCAGCATCTTATCCGAGTGG	TGGATGGTGGTACAGTCAGAGC
*CDKN1A (p21)*	AGGTGGACCTGGAGACTCTCAG	TCCTCTTGGAGAAGATCAGCCG
*ACTB (β-actin)*	CATTGCTGACAGGATGCAGAAGG	TGCTGGAAGGTGGACAGTGAGG
*GAPDH*	GGAGCGAGATCCCTCCAAAAT	GGCTGTTGTCATACTTCTCATGG

## Data Availability

The original contributions presented in this study are included in the article. Further inquiries can be directed to the corresponding authors.

## Supplementary Materials

The following supporting information can be downloaded at: https://www.mdpi.com/article/10.3390/biology15131022/s1, Figure S1: Synergy analysis of the RA+DOX combination in 4T1 cells. Table S1: Combination Design and Viability Data; Table S2: Fraction Affected (Fa) Analysis; Table S3: Combination Index Interpretation.

## Abbreviations

The following abbreviations are used in this manuscript:

ACTB	Actin Beta
ANOVA	Analysis of Variance
ATP	Adenosine Triphosphate
BAX	BCL2-Associated X Protein
BCL2	B-Cell Lymphoma 2
BSA	Bovine Serum Albumin
CASP3	Caspase-3
CASP9	Caspase-9
CI	Combination Index
Ct	Cycle Threshold
DAB	Diaminobenzidine
DCFH-DA	2′,7′-Dichlorodihydrofluorescein Diacetate
DMEM	Dulbecco’s Modified Eagle Medium
DMSO	Dimethyl Sulfoxide
DOX	Doxorubicin
ELISA	Enzyme-Linked Immunosorbent Assay
FBS	Fetal Bovine Serum
FITC	Fluorescein Isothiocyanate
GO	Gene Ontology
GSEA	Gene Set Enrichment Analysis
H_2_O_2_	Hydrogen Peroxide
HRP	Horseradish Peroxidase
IC_50_	Half-Maximal Inhibitory Concentration
KEGG	Kyoto Encyclopedia of Genes and Genomes
MDR	Multidrug Resistance
MTT	3-(4,5-Dimethylthiazol-2-yl)-2,5-Diphenyltetrazolium Bromide
NAC	N-Acetylcysteine
OD	Optical Density
PBS	Phosphate-Buffered Saline
PI	Propidium Iodide
qRT-PCR	Quantitative Reverse Transcription Polymerase Chain Reaction
RA	Rosmarinic Acid
RNA	Ribonucleic Acid
RNase	Ribonuclease
ROS	Reactive Oxygen Species
SD	Standard Deviation
STRING	Search Tool for the Retrieval of Interacting Genes/Proteins
TNBC	Triple-Negative Breast Cancer
TP53	Tumor Protein p53
Triton X-100	Octylphenol Ethoxylate Surfactant
β-actin	Beta Actin

## References

[1] Yin L., Duan J.-J., Bian X.-W., Yu S.-C. (2020). Triple-negative breast cancer molecular subtyping and treatment progress. Breast Cancer Res..

[2] Thankamony A.P., Shikha T. (2023). Phenotypic heterogeneity drives differential disease outcome in a mouse model of triple negative breast cancer. Front. Oncol..

[3] Villegas C., Cortez N., Ogundele A.V., Burgos V., Pardi P.C., Cabrera-Pardo J.R., Paz C. (2024). Therapeutic applications of rosmarinic acid in cancer-chemotherapy-associated resistance and toxicity. Biomolecules.

[4] Huang J.-Y., Hsu T.-W., Chen Y.-R., Kao S.-H. (2024). Rosmarinic acid potentiates cytotoxicity of cisplatin against colorectal cancer cells by enhancing apoptotic and ferroptosis. Life.

[5] Kong C.-Y., Guo Z., Song P., Zhang X., Yuan Y.-P., Teng T., Yan L., Tang Q.-Z. (2022). Underlying the mechanisms of doxorubicin-induced acute cardiotoxicity: Oxidative stress and cell death. Int. J. Biol. Sci..

[6] Tian Y., Lei Y., Wang Y., Lai J., Wang J., Xia F. (2023). Mechanism of multidrug resistance to chemotherapy mediated by P-glycoprotein (Review). Int. J. Oncol..

[7] Zhang Y., Deng H., Zhou H., Lu Y., Shan L., Lee S.M., Cui G. (2019). A novel agent attenuates cardiotoxicity and improves antitumor activity of doxorubicin in breast cancer cells. J. Cell Biochem..

[8] Chaitanya M.V.N.L., Ramanunny A.K., Babu M.R., Gulati M., Vishwas S., Singh T.G., Chellappan D.K., Adams J., Dua K., Singh S.K. (2022). Journey of rosmarinic acid as biomedicine to nano-biomedicine for treating cancer: Current strategies and future perspectives. Pharmaceutics.

[9] Konstantinou E.K., Panagiotopoulos A.A., Argyri K., Panoutsopoulos G.I., Dimitriou M., Gioxari A. (2024). Molecular pathways of rosmarinic acid anticancer activity in triple-negative breast cancer cells: A literature review. Nutrients.

[10] Wang W., Zhang Y., Huang X., Li D., Lin Q., Zhuang H., Li H. (2024). The role of the miR-30a-5p/BCL2L11 pathway in rosmarinic acid-induced apoptosis in MDA-MB-231-derived breast cancer stem-like cells. Front. Pharmacol..

[11] Sarı U., Zaman F. (2024). Effects of rosmarinic acid and doxorubicine on an ovarian adenocarsinoma cell line (OVCAR3) via the EGFR pathway. Acta Cir. Bras..

[12] Toprak V., Özdemir İ., Öztürk Ş., Yanar O., Kizildemir Y.Z., Tuncer M.C. (2024). Modulation of FOXP3 Gene Expression in OVCAR3 Cells Following Rosmarinic Acid and Doxorubicin Exposure. Pharmaceuticals.

[13] Helvacıoğlu S., Hamitoğlu M., Yıldırım E., Vural Korkut Ş., Yaba A., Aydın A. (2025). Protective effects of rosmarinic acid and epigallocatechin gallate against doxorubicin-induced cytotoxicity and genotoxicity in CHO-K1 cells. Turk. J. Pharm. Sci..

[14] Zhao J., Xu L., Jin D., Xin Y., Tian L., Wang T., Zhao D., Wang Z., Wang J. (2022). Rosmarinic acid and related dietary supplements: Potential applications in the prevention and treatment of cancer. Biomolecules.

[15] Kowalczyk A., Tuberoso C.I.G., Jerković I. (2024). The role of rosmarinic acid in cancer prevention and therapy: Mechanisms of antioxidant and anticancer activity. Antioxidants.

[16] Khaksar S., Kiarostami K., Ramdan M. (2024). Effect of rosmarinic acid on cell proliferation, oxidative stress, and apoptosis pathways in an animal model of induced glioblastoma multiforme. Arch. Med. Res..

[17] Bray F., Laversanne M., Sung H., Ferlay J., Siegel R.L., Soerjomataram I., Jemal A. (2024). Global cancer statistics 2022: GLOBOCAN estimates of incidence and mortality worldwide for 36 cancers in 185 countries. CA Cancer J. Clin..

[18] Kciuk M., Gielecińska A., Mujwar S., Kołat D., Kałuzińska-Kołat Ż., Celik I., Kontek R. (2023). Doxorubicin-An agent with multiple mechanisms of anticancer activity. Cells.

[19] Özdemir İ., Baş D.D., Öztürk Ş., Karaosmanoğlu Ö., Tuncer M.C. (2025). Rosmarinic acid inhibits the proliferation of ovarian carcinoma cells by activating the p53/BAX signaling pathway. Histol. Histopathol..

[20] Sur D., Gorzo A., Sabarimurugan S., Krishnan S.M., Lungulescu C.V., Volovat S.R., Burz C. (2022). A comprehensive review of the use of antioxidants and natural products in cancer patients receiving anticancer therapy. Anticancer Agents Med. Chem..

[21] Nunes S., Madureira A.R., Campos D., Sarmento B., Gomes A.M., Pintado M., Reis F. (2017). Therapeutic and nutraceutical potential of rosmarinic acid—Cytoprotective properties and pharmacokinetic profile. Crit. Rev. Food Sci. Nutr..

[22] Al-Hunaiti A., Thiab T.A., Zihlif M., Abdul Majid A.M.S., Imraish A., Batarseh Y., Al Shhab M. (2025). Rosmarinic acid attenuates doxorubicin-induced cardiotoxicity: Bio-nanocarrier system development and an in vitro study using H9c2 rat cardiomyocytes. Nanoscale. Adv..

[23] Green D.R. (2022). The mitochondrial pathway of apoptosis: Part I: MOMP and beyond. Cold Spring Harb. Perspect. Biol..

[24] Reiners J.J., Caruso J.A., Mathieu P., Chelladurai B., Yin X.M., Kessel D. (2002). Release of cytochrome c and activation of pro-caspase-9 following lysosomal photodamage involves Bid cleavage. Cell Death Differ..

[25] Gallyas F., Ramadan F.H.J., Andreidesz K., Hocsak E., Szabo A., Tapodi A., Kiss G.N., Fekete K., Bognar R., Szanto A. (2022). Involvement of mitochondrial mechanisms and cyclooxygenase-2 activation in the effect of desethylamiodarone on 4T1 triple-negative breast cancer line. Int. J. Mol. Sci..

[26] Engeland K. (2022). Cell cycle regulation: P53-p21-RB signaling. Cell Death Differ..

[27] Galluzzi L., Vitale I., Warren S., Adjemian S., Agostinis P., Martinez A.B., Chan T.A., Coukos G., Demaria S., Deutsch E. (2020). Consensus guidelines for the definition, detection and interpretation of immunogenic cell death. J. Immunother. Cancer.

[28] Malik A., Bagchi A.K., Jassal D.S., Singal P.K. (2022). Interleukin-10 mitigates doxorubicin-induced endoplasmic reticulum stress as well as cardiomyopathy. Biomedicines.

[29] Sheikh K.A., Amjad M., Irfan M.T., Anjum S., Majeed T., Riaz M.U., Jassim A.Y., Sharif E.A.M., Ibrahim W.N. (2025). Exploring TGF-β signaling in cancer progression: Prospects and therapeutic strategies. OncoTargets Ther..

[30] Qian S., Wei Z., Yang W., Huang J., Yang Y., Wang J. (2022). The role of BCL-2 family proteins in regulating apoptosis and cancer therapy. Front. Oncol..

[31] Güllülü Ö., Hehlgans S., Rödel C., Fokas E., Rödel F. (2021). Tumor Suppressor Protein p53 and Inhibitor of Apoptosis Proteins in Colorectal Cancer-A Promising Signaling Network for Therapeutic Interventions. Cancers.

[32] Liu Y., Xu X., Tang H., Pan Y., Hu B., Huang G. (2021). Rosmarinic acid inhibits cell proliferation, migration, and invasion and induces apoptosis in human glioma cells. Int. J. Mol. Med..

[33] Mahmoud M.A., Okda T.M., Omran G.A., Abd-Alhaseeb M.M. (2021). Rosmarinic acid suppresses inflammation, angiogenesis, and improves paclitaxel induced apoptosis in a breast cancer model via NF3 κB-p53-caspase-3 pathways modulation. J. Appl. Biomed..

[34] Messeha S.S., Zarmouh N.O., Asiri A., Soliman K.F.A. (2020). Rosmarinic acid-induced apoptosis and cell cycle arrest in triple-negative breast cancer cells. Eur. J. Pharmacol..

[35] Kalniņa Z., Liekniņa I., Koteloviča S., Petrovska R., Žvinys G., Petrosiute A., Zubrienė A., Laugalis M.T., Skeltona V., Jansons J. (2025). Development of 4T1 breast cancer mouse model system for preclinical carbonic anhydrase IX studies. FEBS Open Bio.

[36] De Angelis A., Urbanek K., Cappetta D., Piegari E., Ciuffreda L.P., Rivellino A., Russo R., Esposito G., Rossi F., Berrino L. (2016). Doxorubicin cardiotoxicity and target cells: A broader perspective. Cardiooncology.

[37] Bhutani V., Varzideh F., Wilson S., Kansakar U., Jankauskas S.S., Santulli G. (2025). Doxorubicin-Induced Cardiotoxicity: A Comprehensive Update. J. Cardiovasc. Dev. Dis..

