# Supplementary material for: Effects of Rosmarinic Acid and Doxorubicin Combination in Breast Cancer Cells
Source: Biology (Basel). 2026 Jun 26;15(13):1022. doi: 10.3390/biology15131022 (PMC13360033; doi:10.3390/biology15131022)
Supplement: Supplementary file 1 [file biology-15-01022-s001.zip › biology-4373523-supplementary.pdf]

Supplementary Table S1. Combination Design and Viability Data

RA (µM)	DOX (µM)	RA alone (%)	DOX alone (%)	RA+DOX (%)	Fa	CI
10	1.0	87	68	48	0.52	0.85
20	2.5	75	46	34	0.66	0.77
50	5.0	59	32	26	0.74	0.68
100	10.0	36	22	18	0.82	0.62
250	25.0	24	12	8	0.92	0.57
500	50.0	16	5	4	0.96	0.54

Supplementary Table S2. Fraction Affected (Fa) Analysis

Combination	Viability (%)	Fa
RA10+DOX1	48	0.52
RA20+DOX2.5	34	0.66
RA50+DOX5	26	0.74
RA100+DOX10	18	0.82
RA250+DOX25	8	0.92
RA500+DOX50	4	0.96

Supplementary Table S3. Combination Index Interpretation

Fa	CI	Interpretation
0.52	0.85	Moderate synergism
0.66	0.77	Synergism
0.74	0.68	Synergism
0.82	0.62	Strong synergism
0.92	0.57	Strong synergism
0.96	0.54	Strong synergism

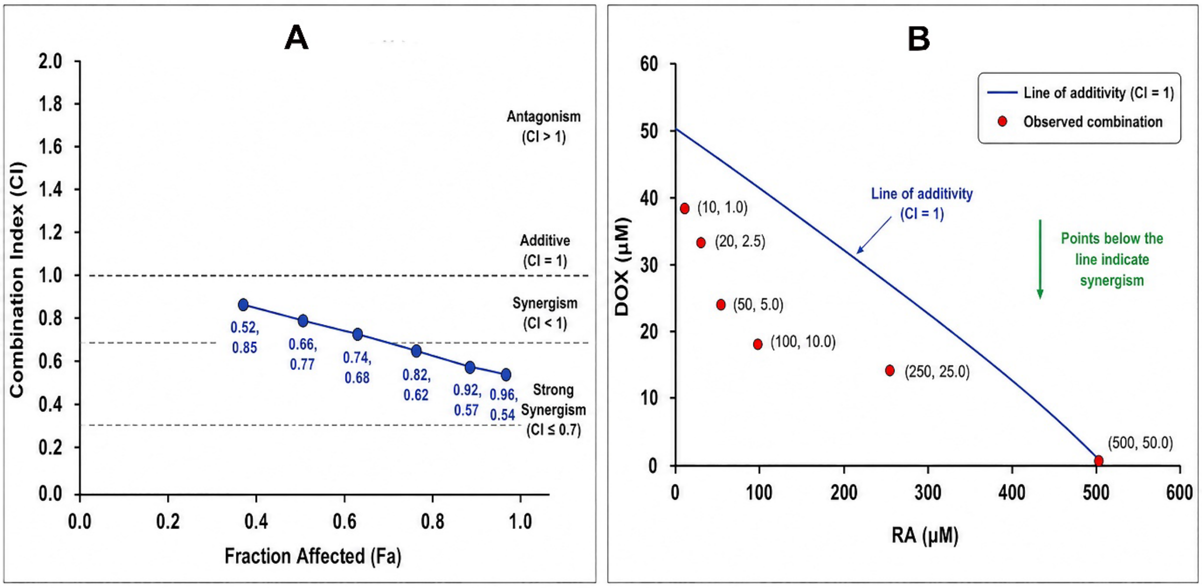

Supplementary Figure S1. Synergy analysis of the RA+DOX combination in 4T1 cells. (A) Fraction affected–Combination Index (Fa–CI) plot generated using the Chou–Talalay method. CI values remained below 1 across all evaluated fraction affected (Fa) levels, indicating synergistic interactions between rosmarinic acid (RA) and doxorubicin (DOX). Increasing Fa values were associated with progressively stronger synergistic effects, with CI values ranging from 0.85 to 0.54. Dashed horizontal lines indicate the thresholds for antagonism (CI > 1), additivity (CI = 1), synergism (CI < 1), and strong synergism (CI ≤ 0.7). (B) Isobologram analysis of the RA+DOX combination. The blue line represents the theoretical line of additivity (CI = 1), whereas red circles represent the experimentally observed combination doses. All evaluated dose combinations were located below the line of additivity, confirming synergistic interactions between RA and DOX in 4T1 cells.
